# The developmental stage of the medulloblastoma cell-of-origin restricts Sonic hedgehog pathway usage and drug sensitivity

**DOI:** 10.1242/jcs.258608

**Published:** 2022-06-08

**Authors:** Marlinde J. Smit, Tosca E. I. Martini, Inna Armandari, Irena Bočkaj, Walderik W. Zomerman, Eduardo S. de Camargo Magalhães, Zillah Siragna, Tiny G. J. Meeuwsen, Frank J. G. Scherpen, Mirthe H. Schoots, Martha Ritsema, Wilfred F. A. den Dunnen, Eelco W. Hoving, Judith T. M. L. Paridaen, Gerald de Haan, Victor Guryev, Sophia W. M. Bruggeman

**Affiliations:** 1 European Research Institute for the Biology of Ageing/ERIBA, University Medical Center Groningen, University of Groningen, Hanzeplein 1, 9700 RB, Groningen, The Netherlands; 2 Department of Pediatrics/Pediatric Oncology and Hematology, University Medical Center Groningen, University of Groningen, Hanzeplein 1, 9700 RB, Groningen, The Netherlands; 3 Glial Cell Biology Laboratory, Biomedical Sciences Institute, Federal University of Rio de Janeiro, Rio de Janeiro, 21949-590, Brazil; 4 Department of Pathology and Medical Biology, University Medical Center Groningen, University of Groningen, Hanzeplein 1, 9700 RB, Groningen, The Netherlands; 5 Princess Máxima Center for Pediatric Oncology, Lundlaan 6, 3584 EA Utrecht, The Netherlands

**Keywords:** Medulloblastoma, Sonic hedgehog signaling, Cerebellar development, Cerebellar granule neuron progenitors, Tumor cell-of-origin, Primary cilia

## Abstract

Sonic hedgehog (SHH) medulloblastoma originates from the cerebellar granule neuron progenitor (CGNP) lineage, which depends on Hedgehog signaling for its perinatal expansion. Whereas SHH tumors exhibit overall deregulation of this pathway, they also show patient age-specific aberrations. To investigate whether the developmental stage of the CGNP can account for these age-specific lesions, we analyzed developing murine CGNP transcriptomes and observed highly dynamic gene expression as a function of age. Cross-species comparison with human SHH medulloblastoma showed partial maintenance of these expression patterns, and highlighted low primary cilium expression as hallmark of infant medulloblastoma and early embryonic CGNPs. This coincided with reduced responsiveness to upstream SHH pathway component Smoothened, whereas sensitivity to downstream components SUFU and GLI family proteins was retained. Together, these findings can explain the preference for *SUFU* mutations in infant medulloblastoma and suggest that drugs targeting the downstream SHH pathway will be most appropriate for infant patients.

## Abstract (precis)

**Summary:** There is a relationship between the age of the medulloblastoma patient and the developmental age of the tumor cell-of-origin, and this influences the SHH pathway signaling route used by the tumor.

## INTRODUCTION

Medulloblastoma is a malignant tumor of the cerebellum that frequently affects children. It consists of four main transcriptional subgroups that can be further subdivided upon additional molecular profiling (Cavalli et al., 2017; Hovestadt et al., 2019; Northcott et al., 2012a, 2017; Vladoiu et al., 2019). The Sonic hedgehog (SHH) subgroup of medulloblastoma, which accounts for 30% of all medulloblastoma cases, is believed to originate from the cerebellar granule neuron progenitor (CGNP) population that critically depends on SHH pathway signaling for its perinatal expansion (Dahmane and Ruiz, 1999; Hovestadt et al., 2019; Salsano et al., 2004; Vladoiu et al., 2019; Wallace, 1999; Wechsler-Reya and Scott, 1999; Yang et al., 2008; Yokota et al., 1996). SHH is a secreted morphogen that controls development and patterning in many organs, including the central nervous system (Fuccillo et al., 2006; Jiang and Hui, 2008). The pathway becomes active when SHH binds to its receptor Patched 1 (PTCH1), which relieves inhibition of Smoothened (SMO) and induces MYCN (Fuccillo et al., 2006; Jiang and Hui, 2008; Kenney, 2003; Knoepfler et al., 2002). Activated SMO inhibits the tumor suppressor protein SUFU and stimulates processing of the GLI factors into transcriptional activators. In the absence of the SHH morphogen, transcription of SHH target genes is actively repressed by SUFU-mediated formation of GLI repressors or direct transcriptional repression (Cheng and Bishop, 2002; Monnier et al., 1998; Pearse et al., 1999).

In line with the above, SHH medulloblastoma is characterized by an overall deregulation of SHH signaling that is often accompanied by mutually exclusive mutations in SHH pathway components, underlining the importance of this pathway in driving tumorigenesis (Kool et al., 2014; Northcott et al., 2012a). Interestingly, there is also patient age-related heterogeneity within this subgroup, suggesting that developmental factors affect tumor biology (Cavalli et al., 2017; Kool et al., 2014; Northcott et al., 2011, 2012b, 2017; Rausch et al., 2012; Wefers et al., 2014). For instance, infant and adult medulloblastoma display distinct gene expression patterns, as well as differences in copy number alterations and tumor localization. Strikingly, mutations in SHH pathway genes are also correlated with patient age (Kool et al., 2014). Whereas *PTCH1* mutations occur across all age groups, *SUFU* mutations are almost exclusively found in infant patients, *GLI2*, *NMYC* and *TP53* mutations in older children, and *SMO* mutations in adults.

These latter findings raise the question of why the pathway is differentially perturbed depending on patient age, as these mutations would presumably have identical outcomes (i.e. enhanced activation of SHH target genes). One explanation is that the tumor cells-of-origin undergo changes in sensitivity to, or usage of, the SHH signaling pathway during development, which would then provoke age-specific oncogenic lesions in the pathway (Jessa et al., 2019). The presumed cell-of-origin for SHH medulloblastoma is part of the CGNP lineage, a highly dynamic cell population that is present over 3 weeks of mouse and 2 years of human development (Carter et al., 2018), with the precise moment of transformation remaining under debate (Gibson et al., 2010; Haldipur et al., 2012; Leto et al., 2016; Lewis et al., 2004; MacHold and Fishell, 2005; Miale and Sidman, 1961; Schüller et al., 2008; Selvadurai et al., 2020; Wang et al., 2005; Wechsler-Reya and Scott, 1999; Yang et al., 2008). In mice, future CGNPs become specified in the upper rhombic lip (uRL) of the hindbrain around embryonic day (E)13.5, from where they migrate across the surface of the cerebellar primordium (Leto et al., 2016; MacHold and Fishell, 2005; Miale and Sidman, 1961; Wang et al., 2005). Here, they form a secondary germinal zone termed external granular layer (EGL), with a peak in proliferation occurring around birth, which is driven by Purkinje neuron-secreted SHH. Once they reach maturity, terminally differentiating granule neurons cease proliferation and migrate inwards until they reach their final destination in the internal granular layer (IGL) of the cerebellum (Dahmane and Ruiz, 1999; Leto et al., 2016; Lewis et al., 2004; Wallace, 1999; Wechsler-Reya and Scott, 1999). Besides the longer gestational period, the development of human CGNPs generally resembles that of the mouse (Haldipur et al., 2012).

To study the potential impact of the CGNP developmental age on medulloblastoma outcome, we have taken advantage of a transgenic mouse model that allows the prospective isolation of the entire CGNP cell lineage during embryonic and postnatal development (Hatten and Roussel, 2011; Machold et al., 2011). As reported before, we confirmed that CGNPs exhibit dynamic changes in gene expression in time, highlighting the identity changes of the CGNP population as neural development progresses (Hovestadt et al., 2019; Machold et al., 2011; Vladoiu et al., 2019). We also confirmed that CGNPs resemble human SHH medulloblastoma samples and, importantly, that early embryonic CGNPs co-segregate with the youngest patients, corroborating a linear relationship between cell-of-origin and patient age. In particular, we found that primary cilium expression was low across young CGNPs and young medulloblastoma patients, which was somewhat unexpected given the importance of primary cilia for SHH pathway activity (Bangs et al., 2015; Chang et al., 2019; Corbit et al., 2005; Haycraft et al., 2005; Huangfu and Anderson, 2005; Huangfu et al., 2003; May et al., 2005; Ong et al., 2020; Rohatgi et al., 2007; Sasai and Briscoe, 2012). In line, early embryonic CGNPs displayed a partial unresponsiveness to SMO-mediated (i.e. upstream) pathway stimulation, preventing increased proliferation. They were, however, sensitive to inhibition of *Sufu* or GLI proteins, both downstream SHH components, which in contrast to SMO, are known to have cilium-independent functions (Chen et al., 2009; Jia et al., 2009). These observations might have clinical implications, as patients with early developmental medulloblastoma are likely to benefit from drugs targeting the downstream primary cilium-independent part of the SHH pathway (Di Magno et al., 2015).

## RESULTS

### Developing CGNPs undergo dynamic changes in the expression of genes implicated in medulloblastoma

**Fig. 1.**
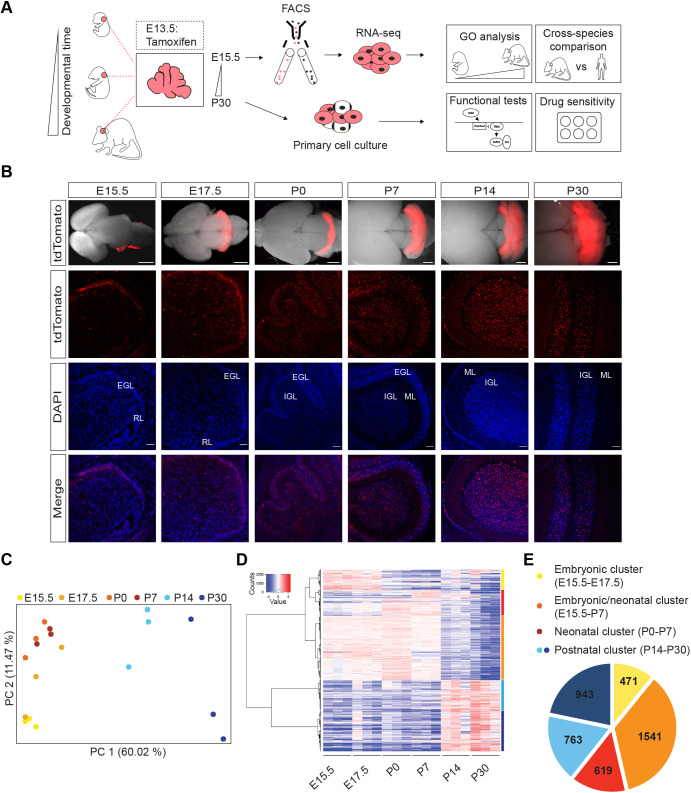
**Developing CGNPs exhibit age-specific gene expression.** (A) Schematic overview of the experimental workflow. To perform transcriptome analysis on developing CGNPs, embryonic E13.5 CGNPs are labeled with tdTomato *in vivo* following a single tamoxifen pulse administered to pregnant females. Fluorescent cerebella are subsequently dissected at different time points between E15.5 and P30, and tdTomato^+^ cells are sorted by FACS. For cell culture experiments, unsorted CGNPs isolated from dissected cerebella are treated with 4-hydroxytamoxifen *in vitro* (E, embryonic day; P, postnatal day; FACS, fluorescence-activated cell sorting). (B) Stereoscopic (top panels) or confocal images (middle and bottom panels) showing native tdTomato fluorescence in whole-mount cerebella and sagittal sections, respectively (RL, rhombic lip; EGL, external granular layer; IGL, internal granular layer; ML, molecular layer). Counterstaining by DAPI. Scale bars: 2 mm (stereoscopic images), 50 μm (confocal images). Images representative of *n*=3 experimental repeats. (C) Principal component analysis of E15.5, E17.5, P0, P7, P14 and P30 CGNP transcriptomes. *n*=3 biological replicates per developmental time point. For embryonic time points, CGNPs from *n*=4 embryos were pooled per sample; for postnatal time points, individual mice were analyzed. See also Table S1. (D) Heatmap showing differentially expressed CGNP genes as a function of developmental time point (unsupervised hierarchical clustering analysis). Genes are clustered according to the branching of the clustering tree into five major clusters: yellow cluster (E15.5–E17.5); orange cluster (E15.5–P7); red cluster (P0–P7); light blue and dark blue clusters (P14–P30). See also Table S2. (E) Pie chart summarizing five major clusters of differentially expressed genes. Yellow cluster (E15.5–E17.5, *n*=471 genes); orange cluster (E15.5–P7, *n*=1541 genes); red cluster (P0–P7, *n*=619 genes); light blue cluster (P14–P30, *n*=763); and dark blue cluster (P14–P30, *n*=943).

To address whether intrinsic changes in the developing medulloblastoma cell-of-origin could contribute to the age-dependent mutations in SHH pathway components found in SHH medulloblastoma, a transgenic mouse model was employed that allows lineage tracing and prospective isolation of the developing murine CGNP cell lineage from their specification in the cerebellar primordium onwards (Fig. 1A) (Feil et al., 1997; Machold et al., 2011; Machold and Fishell, 2005). This transgenic model consisted of the previously published Math1CreER^T2^ transgenic strain (Machold and Fishell, 2005) and a tdTomato reporter transgenic strain Ai14 (Madisen et al., 2010). Accordingly, we observed that treatment of pregnant transgenic mice with a single dose of tamoxifen at E13.5 induced acute and stable labeling of CGNPs with tdTomato in the offspring (Fig. 1B). We dissected fluorescent cerebella at E15.5, E17.5, P0 (day of birth), postnatal day (P)7, P14 and P30, purified tdTomato-expressing CGNPs and generated transcriptomes (Fig. 1A,C–E; Table S1). Subsequent principal component analysis revealed that more than 70% of all variation in gene expression between samples could be explained by developmental age, demonstrating that CGNPs indeed undergo significant changes in gene expression as a function of time (Fig. 1C).

**Fig. 2.**
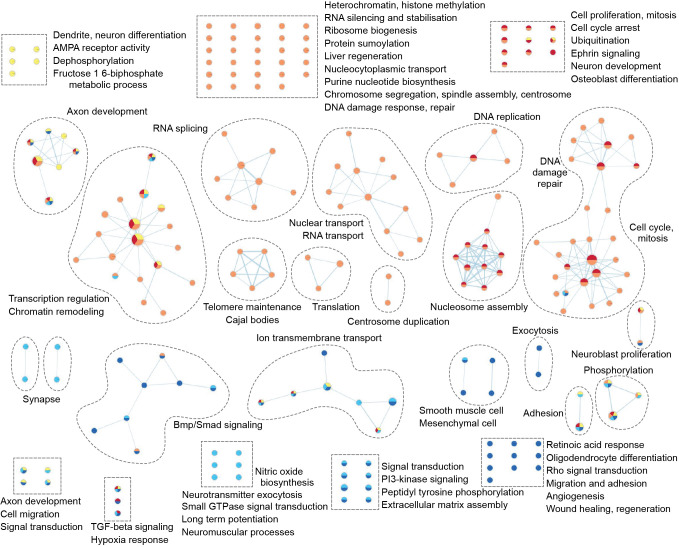
**Specific biological processes are enriched during CGNP development.** Gene ontological analysis shows enriched biological processes in the different CGNP age-specific gene clusters. Each node represents a biological process. Related biological processes are grouped and labeled by biological theme (curved dashed lines). Individual biological processes are assembled in rectangular boxes (dashed lines). Biological processes connected by edges have genes in common. Enriched biological processes were determined with the Database of Annotation, Visualization and Integrated Discovery (DAVID), v.6.8 (Benjamini-corrected q=0.1, *P*=0.01) and visualized with the Enrichment Map app in Cytoscape. Yellow nodes, E15.5–E17.5 cluster; orange nodes, E15.5–P7 cluster; red nodes, P0–P7 cluster; light blue nodes, P14–P30 cluster; dark blue nodes, P14–P30 cluster. See also Fig. S1 and Table S3.

To further explore these dynamic changes, we performed unsupervised hierarchical clustering analysis to identify genes that were differentially expressed (DE) between at least two time points (Fig. 1D; Table S2). We next divided the DE genes into five major gene clusters according to the clustering tree. The yellow cluster contained genes that were highest expressed at embryonic time points (E15.5 and E17.5, *n*=471 genes), the orange cluster genes that were high during embryonic and early postnatal time points (E15.5–P7, *n*=1541 genes), genes in the red cluster were highest expressed at early postnatal time points (P0 and P7, *n*=619 genes), and light and dark blue clusters contained the genes that were induced upon granule neuron maturation (P14 and P30, *n*=763 and 943 genes, respectively) (Fig. 1D,E). To identify enriched biological processes (Gene Ontology) within these gene clusters, we used the Database for Annotation, Visualization and Integrated Discovery (DAVID), and visualized the data using Cytoscape (Fig. 2; Fig. S1, Table S3) (Zomerman et al., 2018). At the early stages of CGNP development (yellow cluster), there is a strong enrichment of biological processes associated with early neural development such as axon and dendrite formation, which consist of genes involved in the attraction and repulsion of migrating progenitor cells, as well as developmental transcription factor-driven gene expression and glycolysis (Fig. 2; Fig. S1). In comparison, processes enriched throughout embryonic and neonatal stages (orange cluster) are biased towards cell proliferation and mitosis. This surge in proliferation might be accompanied by increased DNA damage, as also processes related to the DNA damage response (DDR) are frequent in this cluster. The end of cerebellar proliferation is heralded by the appearance of cell cycle arrest processes, which are specific to early neonatal CGNPs (red cluster). Of note, the vast majority of orange cluster genes show the highest relative gene expression levels at P0, suggesting that proliferation of CGNPs born around E13.5 peaks around birth and declines at P7 (Fig. 1D). Two orange cluster processes (GO terms covalent chromatin modification and ATP-dependent chromatin remodeling) stand out, as genes involved in chromatin regulation like *Arid2*, are known to be mutated in medulloblastoma (Table S3) (Roussel and Stripay, 2018; Zhu et al., 2016). During the final stages of CGNP differentiation (light and dark blue clusters), processes related to neuronal connectivity, differentiation and regeneration are enriched, as well as major signal transduction routes like Bmp, TGF-β and phosphoinositide 3-kinase (PI3K) signaling, which are also known to play a role in medulloblastoma (Angley et al., 2003; Grimmer and Weiss, 2008; Northcott et al., 2012b).

**Fig. 3.**
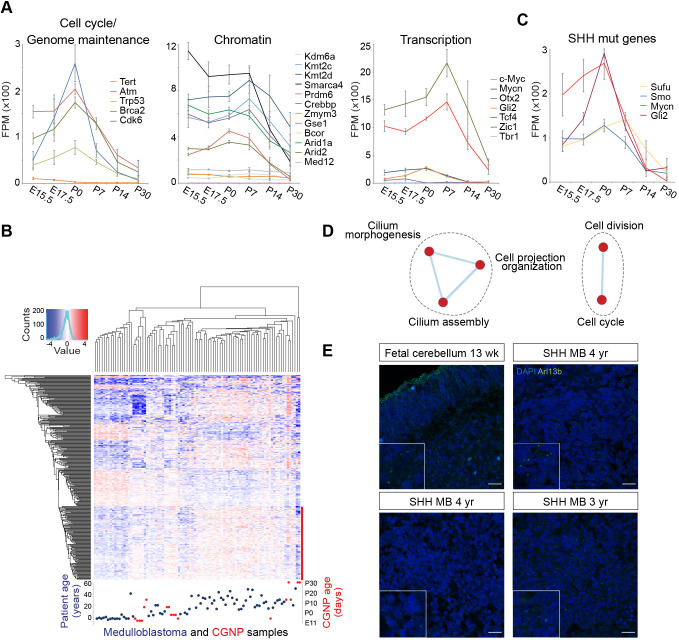
**Cell cycle regulation and primary cilia biogenesis are age-dependent processes in CGNPs and medulloblastoma.** (A) Gene expression profiles of CGNP genes commonly mutated in medulloblastoma, extracted from the RNA-seq data set. Curves represent the average expression level from *n*=3 biological replicates, error bars indicate s.d. (FPM=fragments per million, E, embryonic day; P, postnatal day). See also Fig. S2. (B) Cross-species comparison. Heatmap showing unsupervised hierarchical clustering of differentially expressed human medulloblastoma genes (patient-age groups, 0–3 years, 4–11 years, 12 years and older) and CGNP orthologous genes. Blue dots represent patient samples, red dots represent CGNP samples. Lowest gene cluster indicated by red bar. (C) Gene expression profiles of CGNP genes associated with the SHH signaling pathway, extracted from the RNA-seq data set presented as in A. See also Table S1. (D) Enrichment map representing biological processes enriched in the lower (red) gene cluster of the cross-species comparison heatmap in B. No enriched processes were found in the upper gene clusters. Each node represents a biological process. Related biological processes are grouped and labeled by biological theme (curved dashed lines). Biological processes connected by edges have genes in common. Enriched biological processes were determined with the Database of Annotation, Visualization and Integrated Discovery (DAVID), v.6.8 (Benjamini-corrected q=0.1, *P*=0.01) and visualized with the Enrichment Map app in Cytoscape. (E) Confocal images showing ARL13B protein expression (primary cilium marker) in human fetal cerebellum (upper left panel) and in SHH medulloblastoma samples of 3–4-year-old patients (upper right and lower panels). Counterstaining by DAPI. Images representative of *n*=2 experimental repeats. Insets showing higher magnification (2.25×). Scale bars: 20 µm.

To further explore a potential link between temporal CGNP gene expression and medulloblastoma, we extracted the individual gene expression profiles of *n*=40 genes commonly mutated in medulloblastoma and grouped them as suggested by Northcott et al. (Fig. 3A; Fig. S2; Northcott et al., 2017). We found striking peaks in the expression of the genes involved in cell cycle and genome maintenance at P0, and genes involved in chromatin and transcription regulation at P7, indicating that the age of the cell-of-origin could be related to mutations found in medulloblastoma. Interestingly, we observed the highest induction of *TP53* expression at birth, which is most frequently mutated in children over the age of three (Kool et al., 2014).

### Age-specific CGNP gene expression is preserved in human medulloblastoma

To investigate whether overall CGNP gene expression is reflected in human SHH medulloblastoma, we performed an independent cross-species comparison between an existing cohort of human SHH medulloblastoma and our murine CGNP samples (Fig. 3B) (Kool et al., 2014). For this, we selected the human genes that were differentially expressed between SHH medulloblastoma patients of three age groups, i.e. 0–3 years of age (infants/toddlers), 4–11 years of age (children), and 12 years and older (older children/adults). In agreement with earlier publications, we confirmed that infant and adult medulloblastomas have distinct gene expression patterns, with childhood medulloblastoma forming an intermediate group (Kool et al., 2014; Northcott et al., 2011). Interestingly, unsupervised hierarchical clustering analysis of these human genes together with their murine counterparts showed that embryonic mouse CGNPs co-cluster with the infant medulloblastomas, and older CGNPs with tumors from older patients (Fig. 3B). This demonstrates that CGNPs resemble human medulloblastoma, and that the age of the patient is partially reflected by the age of the medulloblastoma cell-of-origin.

### Cell cycle regulation and primary cilium biogenesis are age-related processes in CGNPs and medulloblastoma

We next re-examined the CGNP transcriptome data with the purpose of finding an explanation for the existence of age-specific SHH pathway mutations. We hereby hypothesized that these genes are differentially susceptible to oncogenic mutation as a function of time because they are not equally important at all stages of development. If true, there could be differences in peak gene expression levels for the mutated SHH pathway genes as we had observed for *Tp53* (Fig. 3A). To test this, we extracted the individual gene expression profiles from genes associated with the SHH signaling pathway from our CGNP transcriptome data. However, although we observed that SHH target genes like *Gli1*, *Gli2*, *Ccnd1*, *Ccnd2*, *Ptch1* and *Ptch2* (*Ccnd1* and *Ccnd2* encode cyclin D1 and D2, respectively), and to a lesser extent *Mycn*, had clear expression peaks at P0, this was not evident for either the *Sufu* or *Smo* genes, which exhibited striking age-specific mutation patterns (Fig. 3C; Fig. S2). Thus, only *Gli2* and *Mycn*, the homologs of which are predominantly mutated in children over the age of three in conjunction with *TP53*, show age-specific gene expression peaks (Kool et al., 2014).

We subsequently searched for alternative age-related processes that could impose differential SHH pathway usage on the CGNPs. For this, we subjected the gene clusters from the cross-species comparison to gene ontological analysis (Fig. 3B,D; Table S4). We identified only five enriched biological processes, which were all enriched in the older CGNP or patient group (Fig. 3B, lower gene cluster indicated by red bar). These processes were either involved in cell proliferation or in primary cilium formation (Fig. 3D; Table S5). Given that it is known that primary cilia are required for SHH signaling, it seemed paradoxical that younger patients have relatively low primary cilia gene expression. We stained a small panel of SHH medulloblastoma samples derived from young patients for ARL13B, a marker for primary cilia (Caspary et al., 2007), and confirmed that there is large variation in the number of ciliated cells between different patients, with some tumors hardly expressing any primary cilia (Fig. 3E) (Gate et al., 2015; Han et al., 2009). Thus, low primary cilium expression can occur in very young SHH medulloblastoma patients.

**Fig. 4.**
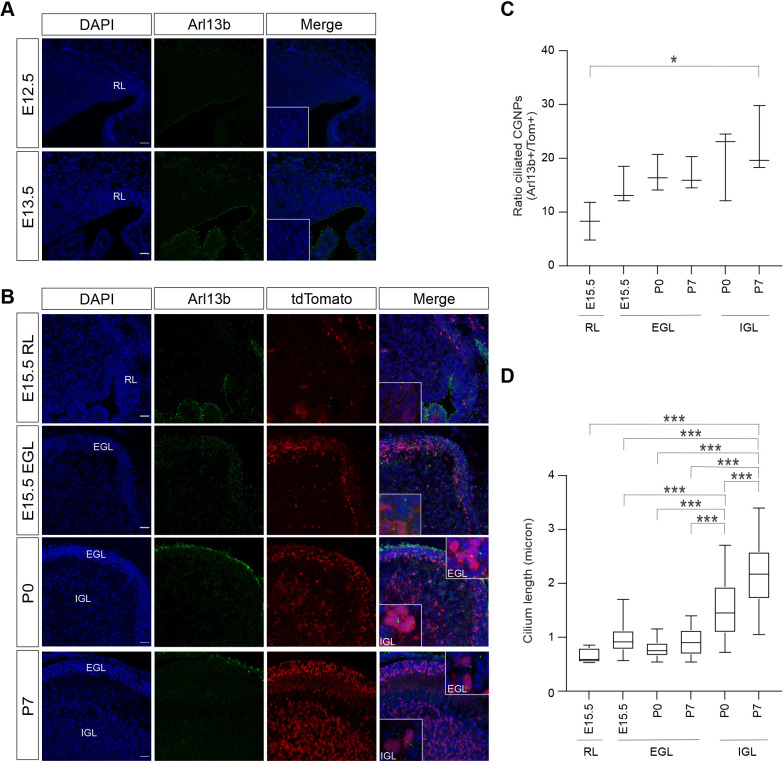
**Primary cilium expression and length in the developing murine cerebellum.** (A) Confocal images showing Arl13b expression in developing mouse cerebellum (E12.5 and E13.5) (RL, rhombic lip; E, embryonic day). Counterstaining by DAPI. Insets showing higher magnification (3.5×). Scale bars: 25 µm. (B) Confocal images showing Arl13b and tdTomato expression in the developing mouse cerebellum (E15.5 RL, E15.5 EGL, P0, P7) (P, postnatal day; RL, rhombic lip; EGL, external granular layer; IGL, internal granular layer). Insets showing higher magnification (5×) . Scale bars: 25 µm. Images in A and B representative of *n*=3 experimental repeats. (C) Plot showing the average ratio of ciliated CGNPs (i.e. Arl13b^+^/tdTomato^+^ cells) per location and developmental timepoint. Results represent median and complete range. *n*=3 biological replicates. (D) Box-and-whisker plot showing the average CGNP primary cilium length per location and developmental timepoint. *n*=3 biological replicates. The box represents the 25–75th percentiles, and the median is indicated. The whiskers show the minimum to maximum. *P<0.05; ***P<0.001 (ordinary one-way ANOVA with Tukey's post-hoc test).

We next investigated primary cilium expression in normal developing CGNPs. In human second trimester fetal cerebellum, we found ciliated cells in both the EGL and IGL, the latter being most prominent (Fig. 3E, upper left panel). As we had no access to first trimester embryonic human cerebellum that harbors the early specified uRL and presumptive EGL cells, we subsequently turned to the developing murine cerebellum to establish the dynamics of primary cilium expression from the uRL stage onwards. In contrast to the general view that most cells have a primary cilium, we found that in the early E12.5–E13.5 mouse cerebellum, cells in the uRL and especially in the future EGL appear to have fewer primary cilia compared to surrounding brain structures (Fig. 4A) (Bangs et al., 2015; Michaud and Yoder, 2006). However, we cannot exclude that some of the apically localized primary cilia contacting the ventricle are expressed by developing CGNPs. Hence, to assess primary cilia expression specifically in developing CGNPs, we reverted to the transgenic mouse model to label Math1-expressing cells and their descendants with tdTomato at E13.5. This permitted quantifying CGNPs carrying a primary cilium from E15.5 onwards. We found that the number of ciliated cells in the EGL and later also the IGL, increases over time (Fig. 4B,C). At neonatal stages, ciliated CGNPs were more abundant towards the outer EGL in agreement with earlier studies (Chang et al., 2019; Ong et al., 2020). However, ciliated CGNPs were most frequent in the IGL, and these cilia were also significantly longer (Fig. 4B–D). Altogether, this demonstrates that both primary cilium expression and length are dynamic during CGNP development, and that at early phases of medulloblastoma cell-of-origin development, primary cilium expression is less pronounced.

### SUFU and SMO expression is dynamic during cerebellar development

**Fig. 5.**
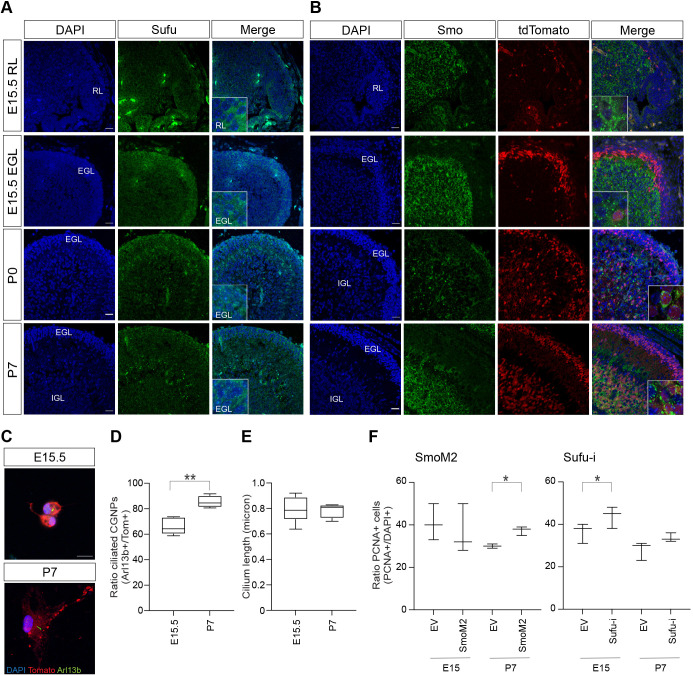
**SUFU and SMO expression and activity in developing CGNPs.** (A,B) Confocal images showing (A) SUFU and (B) Smoothened (SMO) protein expression in the E15.5 RL, E15.5 EGL, P0 and P7 cerebellum. Counterstaining by DAPI (RL, rhombic lip; EGL, external granular layer; IGL, internal granular layer; E, embryonic day; P, postnatal day). Insets showing higher magnification (4×). Scale bars: 25 µm. See also Fig. S3A. (C) Confocal images showing representative examples of Arl13b and tdTomato protein expression in E15.5 (upper panel) and P7 (lower panel) primary CGNP cultures. Counterstaining by DAPI. Scale bar: 10 µm. Images in A–C are representative of *n*=3 experimental repeats. (D,E) Box-and-whisker plots showing primary cilium ratio (D) and length (E) in cultured E15.5 and P7 CGNPs. *n*=4 biological replicates. **P<0.01 (unpaired, two-sided *t*-tests). (F) Plots showing the average ratio of PCNA^+^/DAPI^+^ E15 and P7 cerebellar cells after SmoM2 (left chart) or Sufu-i shRNA (right chart), or empty vector (EV) control retroviral transduction. Results represent median and complete range; *n*=3 biological replicates, except for P7 EV (SmoM2), which was *n*=2. *P<0.05 (for SmoM2, unpaired, two-sided *t*-test; for Sufu-i, paired, two-sided *t*-test). See also Fig. S3B,C. For the box-and-whisker plots, the box represents the 25–75th percentiles, and the median is indicated. The whiskers show the minimum to maximum.

Both *Smo* and *Sufu* function is essential for SHH signaling in the cerebellum, with SMO acting upstream in relaying the signals from SHH morphogen-bound PTCH proteins, and SUFU acting downstream in receiving signals from activated SMO to cease the inhibition of GLI factors (Blaess, 2006; Kim et al., 2011). A major difference though is their dependence on the primary cilium for pathway activation. SMO requires the primary cilium for its function, whereas SUFU is also known to have cilium-independent activity (Chen et al., 2009; Haycraft et al., 2005; Huangfu and Anderson, 2005; Huangfu et al., 2003; Jia et al., 2009; Rohatgi et al., 2007; Spassky et al., 2008). Hence, if a subset of infant medulloblastoma is derived from the earliest specified CGNPs, this could explain the increased frequency of *SUFU* mutations, as these cells are less frequently ciliated. We therefore checked whether during cerebellar development, SUFU and SMO proteins were expressed in patterns consistent with this hypothesis (Fig. 5A,B). In line with cilium-dependent functions, we occasionally detected SUFU and SMO expression in primary cilia (Fig. S3A). Interestingly, SUFU was most prominently expressed in the presumptive EGL at embryonic day E15.5 and was also found in the uRL (Fig. 5A). At postnatal stages, SUFU expression became more restricted towards the outer EGL and upper layers of the IGL. In contrast, SMO expression appeared to be most highly expressed in the area where the IGL forms (Fig. 5B). These differences in expression pattern could indicate differences in activity during cerebellar development.

### Embryonic CGNPs have reduced sensitivity to SMO manipulation but can be activated by downstream SHH signaling

We next set out to functionally test whether embryonic CGNPs are differentially sensitive to SHH pathway component manipulation compared to early postnatal CGNPs, as suggested by the differential expression of SUFU, SMO and primary cilia. For this, we isolated *Math1-CreER^T2^; tdTomato* CGNPs from either E15.5 or P7 cerebellum and cultured them for brief periods of time to preserve their primary status (48–72 h) (Anne et al., 2013; Hatten, 1985; Lee et al., 2009; Salero and Hatten, 2007). We observed ciliated CGNPs in both E15 and P7 cultures (Fig. 5C), suggesting that cilia are not completely absent from E15.5 CGNPs even though they are expressed less frequently (Fig. 5D,E). We then explored SHH pathway activation in these cells. In agreement with earlier publications, we found a significant increase in P7 cell proliferation upon overexpression of oncogenic *Smo* (SmoM2) and a trend towards increased proliferation upon knockdown of *Sufu,* indicating engagement of upstream and downstream SHH pathway segments at this time (Fig. 5F; Fig. S3B,C) (Dahmane and Ruiz, 1999; Kim et al., 2011; Wechsler-Reya and Scott, 1999). However, in E15.5 cells, only *Sufu* knockdown cells showed increased proliferation, whereas SmoM2 overexpression had no effect (Fig. 5F; Fig. S3B,C).

**Fig. 6.**
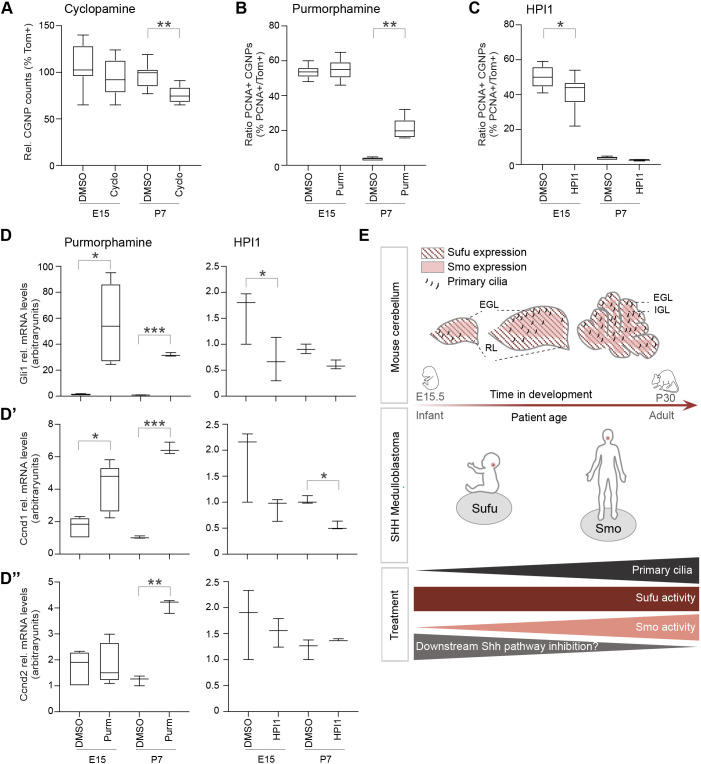
**Embryonic CGNPs are partially insensitive to Smoothened manipulation.** (A) Box-and-whisker plot showing the relative cell counts of tdTomato^+^ (Tom+) CGNPs after 48 h of treatment with DMSO (control) or cyclopamine (5 µM) in E15.5 or P7 CGNP primary cultures. *n*=8 biological replicates. (B) Box-and-whisker plot showing the average ratio of PCNA^+^/tdTomato^+^ cells after 48 h of treatment with DMSO (control) or purmorphamine (250 nM) in E15.5 or P7 CGNP primary cultures. E15, *n*=12; and P7, *n*=5 biological replicates. See also Fig. S3D. (C) Box-and-whisker plot showing the average ratio of PCNA^+^/tdTomato^+^ cells after 48 h of treatment with DMSO (control) or HPI1 (2.5 µM) in E15.5 or P7 CGNP primary cultures. E15, *n*=9; and P7, *n*=5 biological replicates. (D–D″) Results are given in box-and-whisker plots (purmorphamine) or represent median and complete range (HPI1). Plots show relative Gli1 (D), Cyclin D1 (*Ccnd1*) (D′), or Cyclin D2 (*Ccnd2*) (D″) mRNA expression levels compared to *Gapdh* in purmorphamine- (left graphs, 250 nM) or HPI1- (right graphs, 2.5 µM) treated E15.5 and P7 CGNPs, as determined by qRT-PCR. E15 purmorphamine, *n*=5; P7 purmorphamine, *n*=3; E15 HPI1, *n*=3; and P7 HPI1, *n*=3 biological replicates. See also Fig. S3E,F. *P<0.05, **P<0.01, ***P<0.001 (paired, two-sided *t*-test). For the box-and-whisker plots, the box represents the 25–75th percentiles, and the median is indicated. The whiskers show the minimum to maximum. (E) Proposed model. SHH medulloblastoma derives from the CGNP population. CGNPs are specified in the rhombic lip (RL) of the early embryonic cerebellum, from where they migrate across the cerebellar surface to form the external granular layer (EGL). Upon terminal differentiation, CGNPs migrate inwards to form the definitive internal granular layer (IGL). Expression and length of primary cilia, which are important components of upstream SHH signaling, increases as CGNP development progresses. Thus, if oncogenic transformation takes place at late stages of CGNP development, *SMO* may be preferentially mutated as the primary cilia enhance oncogenic SMO activity. However, if CGNP oncogenic transformation occurs at an early embryonic stage, *SUFU* may be preferentially mutated, which controls downstream SHH signaling independently from the primary cilium. This implies that targeted therapy for infant SHH medulloblastoma should be directed towards downstream tumor-driving mechanisms.

Prompted by these results, we wanted to address whether E15.5 CGNPs are sensitive to treatment with the SMO inhibitor cyclopamine, derivatives of which are being used in the treatment of relapsed SHH medulloblastoma (Taipale et al., 2000). Whereas total cell counts of P7 CGNPs were reduced by cyclopamine treatment as was shown previously by others, E15.5 CGNPs appeared less sensitive (Fig. 6A) (Kenney, 2003; Lafranchi et al., 2014). Likewise, treatment with purmorphamine, a SMO agonist, had no effect on E15.5 CGNP proliferation whereas P7 CGNPs were highly sensitive (Fig. 6B; Fig. S3D). In contrast, when we treated E15.5 CGNPs with HPI1, a compound that inhibits the downstream SHH target GLI1, we did see a significant reduction in proliferation, implying that the downstream SHH pathway is functional in early embryonic CGNPs (Fig. 6C). To further address this at the molecular level, we analyzed the expression of key SHH target genes (e.g. *Gli1*, *Ptch1*, *Nmyc*, *Ccnd1* and *Ccnd2*) in purmorphamine- or HPI1-treated E15.5 and P7 CGNPs (Fig. 6D–D″; Fig. S3E,F). Surprisingly, whereas E15.5 CGNPs did not increase proliferation upon purmorphamine treatment, they did show significant induction of *Gli1* gene expression, suggesting that these early cells are not completely unresponsive to upstream SHH signaling (Fig. 6D). However, in line with the absence of a proliferative response, they did not increase expression of *Ccnd2* as did P7 CGNPs (Fig. 6D″). Together, these data show that the age of the medulloblastoma cell-of-origin determines the extent of the response to SHH pathway activation, and as a consequence has an impact on SHH drug sensitivity (Fig. 6E).

## DISCUSSION

Whereas SHH medulloblastoma is characterized by an overall deregulation of the SHH signaling pathway, there are also abnormalities specific to patient age (Gibson et al., 2010; Kho et al., 2004; Kool et al., 2014; Northcott et al., 2011; Northcott et al., 2017). In this study, we set out to address whether intrinsic changes in developmental processes between young and older CGNPs, a cell lineage known to be highly susceptible to SHH medulloblastoma formation, can account for the age-specific characteristics of this tumor type (Hatten and Roussel, 2011; Yang et al., 2008).

### The developmental age of the medulloblastoma cell-of-origin is partially reflected in tumors

The idea that naturally occurring changes in identity of the tumor cell lineage-of-origin impact on tumor outcome, is supported by studies showing differences in tumor onset and phenotype in relation to timing of tumor induction, although the type of genetic lesion and intra-tumoral heterogeneity are also expected play a role (Dey et al., 2012; Gibson et al., 2010; Hovestadt et al., 2019; Ohli et al., 2015; Swartling et al., 2012; Tan et al., 2018; Vladoiu et al., 2019). Indeed, when we compared gene expression patterns between developing CGNPs, we found unique enriched biological processes at each developmental stage, and several of those have been linked to SHH medulloblastoma. A linear chronological relationship between CGNP and patient age was evident in some but not all cases (Cavalli et al., 2017; Kho et al., 2004; Kool et al., 2014; Northcott et al., 2011; Northcott et al., 2017). For instance, neurotransmission activity and neural development are enriched in both early embryonic CGNPs and infant medulloblastoma (Cavalli et al., 2017). PI3K signaling, on the other hand, which is also associated with infant medulloblastoma, is mostly enriched in the late postnatal CGNP samples representing maturing granule neurons (Kool et al., 2014). Intriguingly, there was a strong enrichment for processes related to cell cycle control and the DNA damage response throughout early CGNP development with a noticeable peak around birth. As these processes are overrepresented within the older SHH medulloblastoma subtypes, this suggests that the SHH-induced perinatal surge in CGNP proliferation, possibly coinciding with increased DNA damage due to replication stress, is a critical event during cerebellar development that brings along the risk of developing child and adulthood medulloblastoma (Cavalli et al., 2017; Hovestadt et al., 2019; Northcott et al., 2017; Vladoiu et al., 2019). The strong increase in *TP53* expression at birth is particularly interesting, as it might be related to the high incidence of *TP53* mutations and occurrence of chromothripsis in children (Rausch et al., 2012).

### Differential SHH pathway regulation during cerebellar development might impact on infant SHH medulloblastoma outcome

An important remaining question is the precise nature of the cell-of-origin for infant SHH medulloblastoma. In our cross-species comparison, we found that the infant SHH medulloblastomas clustered towards the earliest embryonic CGNPs, suggesting an early embryonic granule neuron progenitor (up to E15.5) as the cell-of-origin (Gibson et al., 2010). This is in line with elegant single-cell sequencing studies, which showed that a subset of SHH medulloblastoma clustered with early embryonic cerebellar cells that were identified by the authors as CGNPs and unipolar brush cell precursors of comparable age (Hovestadt et al., 2019; Vladoiu et al., 2019). However, initially we were puzzled by the fact that both infant SHH tumors and early embryonic CGNPs were characterized by low primary cilium expression, as the latter is believed to be indispensable for SHH signaling (Huangfu et al., 2003; Sasai and Briscoe, 2012; Wheway et al., 2018); how can we reconcile early embryonic CGNPs being the cell-of-origin for infant medulloblastoma exhibiting deregulated SHH signaling, if these cells are not capable of SHH signaling? And moreover, how can we then explain that there is a higher incidence of *SUFU* mutations, and complete lack of *SMO* mutations, in these tumors (Kool et al., 2014)?

We now think that the unique role of SUFU in the SHH pathway might be the answer to these questions. It had already been shown that in contrast to *Smo*, *Sufu* does not absolutely depend on the primary cilium to exert its control over SHH target gene expression (Chen et al., 2009; Corbit et al., 2005; Jia et al., 2009; Rohatgi et al., 2007). In addition, another vital difference between *Smo* and *Sufu* is their effect on SHH signaling, as SMO activates and SUFU represses target gene expression (Cheng and Bishop, 2002; Fuccillo et al., 2006; Jiang and Hui, 2008; Kenney, 2003; Knoepfler et al., 2002). We therefore favor a model in which transformation of early stage CGNPs into infant medulloblastoma requires *SUFU* deletion, as this would induce precocious pathway activation independently of a SHH signal, but not SMO activation, which would not have a proliferative effect, possibly as the result of less frequent primary cilium expression (Han et al., 2009). This idea is supported by studies from others showing a significantly earlier role in cerebellar development for *Sufu* compared to *Smo* and *Shh* (Blaess, 2006; Corrales, 2004; Corrales, 2006; Kim et al., 2011; Lewis et al., 2004; Svärd et al., 2006; Varjosalo et al., 2006). Furthermore, mutant cerebella lacking primary cilia also show relatively late developmental phenotypes, in line with primary cilia being most important during perinatal SHH-induced CGNP proliferation (Chizhikov et al., 2007; Spassky et al., 2008). Whether the frequency or maturity the primary cilium itself plays a functional role in this, awaits further investigation.

### Implications for future medulloblastoma research

Only recently, it was demonstrated that the four consensus subgroups of medulloblastoma can be further subclassified using a combination of molecular profiling techniques (Cavalli et al., 2017; Kool et al., 2014; Lin et al., 2016; Northcott et al., 2011; Northcott et al., 2017). These studies have provided a wealth of information on the molecular genetics, as well as putative regions of origin for these tumors, which are both essential pieces of information for developing accurate preclinical models and subsequential drug testing. For instance, although several mouse models for medulloblastoma have been generated, none of them has faithfully recapitulated infant SHH medulloblastoma, suggesting that the correct cell-of-origin has not been properly targeted (Pöschl et al., 2014). The importance of bona fide preclinical modeling is underscored by our finding that early embryonic CGNPs, the putative cells-of-origin for infant SHH medulloblastoma, are less sensitive to SMO inhibition. Thus, in addition to taking into account the level at which the SHH pathway is compromised when designing targeted therapy, intrinsic characteristics of the cell-of-origin preserved in the tumor should also be taken into consideration (Hassounah et al., 2012; Zhao et al., 2017). Especially in infants, who typically do not receive radiotherapy, it is crucial to use drugs precisely tailored to their specific medulloblastoma subtype (Sharpe et al., 2015).

## MATERIALS AND METHODS

### Experimental animals

The *Math1-CreER^T2^; tdTomato* compound transgenic mouse strain was derived from the Math1CreER^T2^ (Machold and Fishell, 2005) and Ai14 mouse strains (Madisen et al., 2010) (The Jackson Laboratory, strains #007684 and #007914), and was in a C57BL6/mixed background. Mice were conventionally housed, fed *ad libitum* and routinely genotyped by PCR. Timed matings were performed overnight, with the following morning considered E0.5.

Pregnancies were detected by measuring female weight gain at E13.5. A subset of pregnant females received a single dose of tamoxifen (2 mg/100 μl peanut oil, Sigma) by oral gavaging at E13.5. Pregnant females were killed by asphyxiation (CO_2_), neonatal mice until the age of P7 were killed by decapitation. Offspring from different gender were randomly assigned. All animal experiments were approved by the Institutional Animal Care and Use Committee of the University Medical Center Groningen, The Netherlands.

### CGNP single-cell suspension preparation

For transcriptional analyses, CGNPs were harvested from E15.5, E17.5, P0, P7, P14 and P30 tdTomato^+^ dissected cerebella from offspring from tamoxifen-treated females. For primary cell cultures, CGNPs were harvested from E15.5 and P7 days old cerebella from offspring of non-treated females. Cerebellar dissection was performed using a stereomicroscope. E15–P30 cerebella were dissociated with a papain dissociation kit following the manufacturer's instructions (Worthington). Following papain treatment, ovomucoid was added to stop the reaction. For P14 and P30 cerebella, an additional Percoll gradient step was performed to remove myelin and debris. Cell suspensions were filtered through a 40 μm cell strainer prior to further processing.

### Primary CGNP cell cultures

For embryonic CGNP cultures (E15.5), *n*=8–20 dissected cerebella were pooled. For P7 CGNP cultures, *n*=2–4 dissected cerebella were pooled. Single-cell suspensions were prepared as described above. Cells were pelleted and resuspended in the appropriate culture media. For E15.5 CGNPs, this was Basal medium Eagle (BME) supplemented with 1% N2, 2% B27 (Invitrogen) and 0.25 µg ml^−1^ SHH (R&D systems). For P7 CGNPs, Dulbecco's modified Eagle's medium and Ham's F-12 (DMEM-F12) supplemented with 1% N2, 2% B27, 1.5% sucrose, 5 μm HEPES (Invitrogen) and 0.25 µg ml^−1^ SHH (R&D systems) was used. For proliferation assays, 1 µM 4-hydroxytamoxifen (Sigma) was added to the culture media. For proliferation assays and RNA isolation, 250,000 E15.5 CGNPs or 500,000 P7 CGNPs cells were seeded into poly-D-lysine (100 µg ml^−1^, Sigma)-coated 24-well plates (E15.5) or 12-well plates (P7). For confocal imaging, 100,000 E15.5 CGNPs or 60,000 P7 CGNPs were seeded onto Ibidi 8-chamber glass slides coated with poly-D-lysine.

For drug treatments, E15.5 or P7 CGNPs were plated as described above in the presence 5 µM cyclopamine (Merck), 250 nM purmorphamine (Stem Cell Technologies), 2.5 µM HPI1 (Tocris) or DMSO, and incubated for 48 h. Cells were seeded as one or more (if sufficient cells were available) replicates per experiment, and at least three biological replicates (e.g. samples derived from different mice) were assayed as indicated in the figure legends.

### Lentiviral transductions

The *Sufu* shRNA construct was generated by cloning a Sufu-targeting 22-mer oligonucleotide into a modified pRRL-SFFV-IRES-GFP plasmid (restriction sites XhoI and EcoRI), with tNGFR replaced by GFP (kind gift from Dr Hein Schepers, Dept of Cell Biology, University Medical Center Groningen, The Netherlands; see also Table S6) (Korthuis et al., 2015).

293T producer cells (ATCC, routinely tested free from mycoplasm) were transfected with prrl-SFFV-IRES-GFP, prrl-SFFV-IRES-GFP-Sufu or SmoM2 (W535L)-pcw107-V5 (Addgene #64628) using Fugene HD transfection reagent (Promega). Appropriate CGNP culture media supplemented with B27 was added to the producer cells 16 h prior to virus harvest. CGNPs were subsequently incubated with lentiviruses for 2 h, after which virus was gradually replaced with culture medium to prevent cell death. Cells were fixed at 48 h following transduction.

### Quantification of cells and primary cilia

For quantification of PCNA-positive CGNPs in primary cultures, random fluorescence microscopic images (*n*=10 per sample) were taken and processed using CellProfiler 3.1.9 software (Broad Institute, cellprofiler.org) using an adapted Counting and Scoring pipeline. Average ratios of double positive cells (PCNA+/tdTomato+) per field of view were calculated and tested for significance (*P*<0.05), as stated in the figure legend.

For quantification of total CGNP numbers after cyclopamine treatment, the average number of tdTomato-positive cells per field of view was determined using Cell Profiler. Cell counts were subsequently normalized to EV or DMSO control.

For cells lentivirally transduced with SmoM2 or Sufu shRNA (prrl-SFFV-IRES-GFP-Sufu), the PCNA+/DAPI+ fraction was counted manually using Fiji software.

For primary cilium quantification or length measurement, ARL13B and tdTomato (double) positive cells were counted manually per developmental time point in cultured CGNPs, or in RL, EGL and IGL, using confocal imaging and Fiji software. Average ratios of double-positive cells per field of view were determined and tested for significance as described in the figure legend.

### Tissue treatments and immunofluorescence

CGNPs were fixed with 100% methanol (for PCNA staining) or 4% formaldehyde (for ARL13B), and blocked with 0.1% Triton X-100, 1% bovine serum albumin (BSA) and 0.05% Tween 20 in PBS. Primary antibodies used were against: ARL13B (Proteintech, 17711-1-AP, lot #00049885, 1:400), PCNA (Abcam, ab29, lot #GR3195972-1, 1:1000) and RFP (Rockland, 600-401-379, lot #36233, 1:500). Secondary antibodies were Alexa Fluor 488 (1:500) and Alexa Fluor 568 (1:500) conjugated Invitrogen). Cells were counterstained with DAPI (Sigma).

Brains from tamoxifen-treated Math1-CreER^T2^; tdTomato mice were dissected from the skull, fixed with 4% formaldehyde, and cryoprotected with a sucrose gradient (10%, 20% and 30% sucrose in PBS). They were embedded into Tissue-Tek O.C.T. compound (Sakura-FineTek) and snap frozen in liquid nitrogen. Human fetal brain and medulloblastoma tissue was obtained through the University Medical Center Groningen, The Netherlands and the Princess Máxima Center for Pediatric Oncology, Utrecht, The Netherlands. Tumor samples and clinical data were provided by collaborating centers with approval from the respective institutional review boards/ethics committees [Medical Ethics Review Board (METc) UMCG Groningen, and the Medical Ethics Review Board (METc) Utrecht] in agreement with the Declaration of Helsinki; and informed consent from all patients or their guardians.

Cryosections were generated on a Leica cryostat. Paraffin sections were deparaffinized and rehydrated prior to further processing. Antigen retrieval was performed by boiling tissue sections in citrate buffer (100 mM, pH 6.0), except for ARL13B and mCherry. Sections were blocked with 5% normal goat serum and 0.1% Triton X-100 (Cell Signaling) in PBS. Primary antibodies used were against: ARL13B (Proteintech, 17711-1-AP, lot #00049885, 1:100), ARL13B (for co-staining with SUFU or SMO; Abcam, ab136648, lot #GR3394308-1, 1:100), mCherry (SICGEN, AB0040-200, lot #0081030119, 1:200), PCNA (Abcam, ab29, lot #GR3195972-1, 1:1000), RFP (Rockland, 600-401-379, lot# 36233, 1:500), SUFU (Abcam, ab28083, lot #GR3263748-3, 1:50), and SMO (Abcam, ab38686, lot #GR198520-1, 1:500). Secondary antibodies were Alexa Fluor 488 (1:500) and Alexa Fluor 568 (1:500) conjugated (Invitrogen). Slides were counterstained with DAPI (Sigma), and mounted with Vectashield (Vector Laboratories) or Prolong Diamond Antifade Mountant (Thermo Scientific).

### Microscopy

Whole-mount images of fluorescent embryonic and postnatal mouse brain were made on an Olympus stereozoom SZX-16 microscope. Primary cells were imaged on an EVOS FL inverted fluorescence microscope (Life Technologies). Fluorescent tissue sections were imaged on Leica TCS SP8 or SP8X DLS confocal microscopes, and analyzed using Fiji (Schindelin et al., 2012).

### Quantitative RT-PCR

RNA from E15.5 and P7 CGNPs was isolated using the RNeasy Mini Kit (Qiagen). cDNA was synthesized using random hexamer primers, dNTPs and Ribolock (Thermo Fisher Scientific). Reverse transcription was performed using reverse transcriptase (Thermo Fisher Scientific). Quantitative RT-PCR was performed on a LightCycler 480 System (Roche) system using universal SYBR^®^ Green supermix (Bio-Rad). Relative gene expression was calculated using the 2^−ΔΔ^CT method. Expression levels were normalized to housekeeping gene *Gapdh*. For primers, see Table S6.

### CGNP FACS and RNA isolation for RNA-Seq

Cerebellar single-cell suspensions (E15.5–P30) were subjected to fluorescence-activated cell sorting (FACS) for tdTomato^+^ cells, using a MoFlo Astrios cell sorter (Beckman Coulter). tdTomato^+^ cells (CGNPs) were collected in 1.5 ml tubes, centrifuged (500 ***g***, 10 min at 4°C) and snap frozen. For the E15.5 and E17.5 time points, tdTomato-positive cerebella from *n*=4–8 embryos from the same litter were pooled prior to FACS to constitute one biological replicate; for P0–P30 time points, individual mice were sorted to constitute a biological replicate.

RNA was extracted using the NucleoSpin RNA XS kit (Macherey-Nagel), following the manufacturer's instructions. RNA concentration was measured with a QuBit RNA HS assay kit (Thermo Fisher Scientific) and analyzed on a bioanalyzer using the Agilent RNA 6000 Pico Kit (Agilent). 2.5–10 ng of RNA per sample was used as input for library preparation.

### Library preparation and RNA-seq

For library prepping, the Clontech SMARTer stranded total RNA-seq kit-pico was used according to the manufacturer's instructions for mammalian sample input. Three biological replicates were sequenced for each time point (Illumina HiSeq 2500). On average, 16.7 million reads (63 bp single-end) were generated for each replicate. Reads were aligned to the mouse reference genome (GRCm38 assembly, gene annotation from Ensembl release 84, http://www.ensembl.org) and quantified using STAR 2.5.3a (Dobin et al., 2013).

### Hierarchical clustering analysis and gene expression analysis

For hierarchical clustering analysis, differentially expressed genes were called for all possible pairwise comparisons of developmental stages (three replicates each) using the edgeR package (Robinson et al., 2010). Genes were selected that significantly changed their expression [false discovery rate (FDR)<10^−5^] in one or more comparisons. Log2 ratios were calculated relative to the average FPM expression of a gene. Unsupervised hierarchical clustering and heatmap plotting was performed using the gplots library (https://github.com/talgalili/gplots). The Canberra distance and Ward clustering method was used.

For assessing individual gene expression, *n*=40 selected genes described in Northcott et al. (Northcott et al., 2017), or genes related to the SHH signaling pathway, were extracted from the RNA-seq data set (Table S1), normalized to fragments per million, and plotted using Excel.

### Cross species comparison

For comparison of gene expression profiles between mouse CGNP and human medulloblastoma patients, a published human medulloblastoma data set was downloaded from GEO (accession number GSE49243). Expression values were transformed into log2 fold change (compared to average expression of a gene across all patients). Unambiguous orthologs (one to one orthology) were determined using the Ensembl Biomart tool (http://www.ensembl.org/biomart). Unsupervised hierarchical clustering and heatmap plotting was performed using the gplots library. The Euclidean distance and average clustering method was used.

### Gene ontology

Pathway enrichment analysis was performed by uploading lists of differentially expressed genes (gene clusters), to the Database for Annotation, Visualization and Integrated Discovery v6.8 (DAVID), and subsequent analysis for gene ontology of biological processes was performed (Huang et al., 2009). Lists of biological processes were imported into the Enrichmentmap app in Cytoscape v3.2.1 (Merico et al., 2010; Shannon et al., 2003). Biological processes were Benjamini-corrected with a moderately permissive q value of <0.1 and a *P*-value of <0.01. Enrichment maps represent biological processes enriched in the gene clusters. Each node represents a biological process grouped and labeled by biological theme. Biological processes connected by edges have genes in common using a Jaccard and Overlap coefficient combined with a similarity cutoff value of 0.375.

## Supplementary Material

Supplementary information

## References

[JCS258608C1] Angley, C., Kumar, M., Dinsio, K. J., Hall, A. K. and Siegel, R. E. (2003). Signaling by bone morphogenetic proteins and smad1 modulates the postnatal differentiation of cerebellar cells. *J. Neurosci.* 23, 260-268. 10.1523/JNEUROSCI.23-01-00260.2003 12514223 PMC6742155

[JCS258608C2] Anne, S. L., Govek, E.-E., Ayrault, O., Kim, J. H., Zhu, X., Murphy, D. A., Van Aelst, L., Roussel, M. F. and Hatten, M. E. (2013). WNT3 inhibits cerebellar granule neuron progenitor proliferation and medulloblastoma formation via MAPK activation. *PLoS One* 8, e81769. 10.1371/journal.pone.0081769 24303070 PMC3841149

[JCS258608C3] Bangs, F. K., Schrode, N., Hadjantonakis, A. K. and Anderson, K. V. (2015). Lineage specificity of primary cilia in the mouse embryo. *Nat. Cell Biol.* 17, 113-122. 10.1038/ncb3091 25599390 PMC4406239

[JCS258608C4] Blaess, S. (2006). Sonic hedgehog regulates Gli activator and repressor functions with spatial and temporal precision in the mid/hindbrain region. *Development* 133, 1799-1809. 10.1242/dev.02339 16571630

[JCS258608C5] Carter, R. A., Bihannic, L., Rosencrance, C., Hadley, J. L., Tong, Y., Phoenix, T. N., Natarajan, S., Easton, J., Northcott, P. A. and Gawad, C. (2018). A single-cell transcriptional atlas of the developing murine cerebellum. *Curr. Biol.* 28, 2910-2920.e2. 10.1016/j.cub.2018.07.062 30220501

[JCS258608C6] Caspary, T., Larkins, C. E. and Anderson, K. V. (2007). The graded response to sonic hedgehog depends on cilia architecture. *Dev. Cell* 12, 767-778. 10.1016/j.devcel.2007.03.004 17488627

[JCS258608C7] Cavalli, F. M. G., Remke, M., Rampasek, L., Peacock, J., Shih, D. J. H., Luu, B., Garzia, L., Torchia, J., Nor, C., Morrissy, A. S. et al. (2017). Intertumoral heterogeneity within medulloblastoma subgroups. *Cancer Cell* 31, 737-754.e6. 10.1016/j.ccell.2017.05.005 28609654 PMC6163053

[JCS258608C8] Chang, C. H., Zanini, M., Shirvani, H., Cheng, J. S., Yu, H., Feng, C. H., Mercier, A. L., Hung, S. Y., Forget, A., Wang, C. H. et al. (2019). Atoh1 controls primary cilia formation to allow for SHH-triggered granule neuron progenitor proliferation. *Dev. Cell* 48, 184-199.e5. 10.1016/j.devcel.2018.12.017 30695697

[JCS258608C9] Chen, M. H., Wilson, C. W., Li, Y. J., Law, K. K. L., Lu, C. S., Gacayan, R., Zhang, X., Hui, C. C. and Chuang, P. T. (2009). Cilium-independent regulation of Gli protein function by Sufu in Hedgehog signaling is evolutionarily conserved. *Genes Dev.* 23, 1910-1928. 10.1101/gad.1794109 19684112 PMC2725943

[JCS258608C10] Cheng, S. Y. and Bishop, J. M. (2002). Suppressor of Fused represses Gli-mediated transcription by recruiting the SAP18-mSin3 corepressor complex. *Proc. Natl. Acad. Sci. U. S. A* 99, 5442-5447. 10.1073/pnas.082096999 11960000 PMC122788

[JCS258608C11] Chizhikov, V. V., Davenport, J., Zhang, Q., Shih, E. K., Cabello, O. A., Fuchs, J. L., Yoder, B. K. and Millen, K. J. (2007). Cilia proteins control cerebellar morphogenesis by promoting expansion of the granule progenitor pool. *J. Neurosci* 27, 9780-9789. 10.1523/JNEUROSCI.5586-06.2007 17804638 PMC6672978

[JCS258608C12] Corbit, K. C., Aanstad, P., Singla, V., Norman, A. R., Stainier, D. Y. R. and Reiter, J. F. (2005). Vertebrate Smoothened functions at the primary cilium. *Nature* 437, 1018-1021. 10.1038/nature04117 16136078

[JCS258608C13] Corrales, J. D. (2004). Spatial pattern of sonic hedgehog signaling through Gli genes during cerebellum development. *Development* 131, 5581-5590. 10.1242/dev.01438 15496441

[JCS258608C14] Corrales, J. D. (2006). The level of sonic hedgehog signaling regulates the complexity of cerebellar foliation. *Development* 133, 1811-1821. 10.1242/dev.02351 16571625

[JCS258608C15] Dahmane, N. and Ruiz, A. (1999). Sonic hedgehog regulates the growth and patterning of the cerebellum. *Development* 3100, 3089-3100. 10.1242/dev.126.14.3089 10375501

[JCS258608C16] Dey, J., Ditzler, S., Knoblaugh, S. E., Hatton, B. A., Schelter, J. M., Cleary, M. A., Mecham, B., Rorke-Adams, L. B. and Olson, J. M. (2012). A distinct smoothened mutation causes severe cerebellar developmental defects and medulloblastoma in a novel transgenic mouse model. *Mol. Cell. Biol.* 32, 4104-4115. 10.1128/MCB.00862-12 22869526 PMC3457348

[JCS258608C17] Di Magno, L., Coni, S., Di Marcotullio, L. and Canettieri, G. (2015). Digging a hole under Hedgehog: Downstream inhibition as an emerging anticancer strategy. *Biochim. Biophys. Acta Rev. Cancer* 1856, 62-72. 10.1016/j.bbcan.2015.06.003 26080084

[JCS258608C18] Dobin, A., Davis, C. A., Schlesinger, F., Drenkow, J., Zaleski, C., Jha, S., Batut, P., Chaisson, M. and Gingeras, T. R. (2013). STAR: Ultrafast universal RNA-seq aligner. *Bioinformatics* 29, 15-21. 10.1093/bioinformatics/bts635 23104886 PMC3530905

[JCS258608C19] Feil, R., Wagner, J., Metzger, D. and Chambon, P. (1997). Regulation of Cre recombinase activity by mutated estrogen receptor ligand-binding domains. *Biochem. Biophys. Res. Commun.* 237, 752-757. 10.1006/bbrc.1997.7124 9299439

[JCS258608C20] Fuccillo, M., Joyner, A. L. and Fishell, G. (2006). Morphogen to mitogen: the multiple roles of hedgehog signalling in vertebrate neural development. *Nat. Rev. Neurosci.* 7, 772-783. 10.1038/nrn1990 16988653

[JCS258608C21] Gate, D., Danielpour, M., Bannykh, S. and Town, T. (2015). Characterization of cancer stem cells and primary cilia in medulloblastoma. *CNS Neurol. Disord. Drug Targets* 14, 600-611. 10.2174/1871527314666150429113851 25921740

[JCS258608C22] Gibson, P., Tong, Y., Robinson, G., Thompson, M. C., Currle, D. S., Eden, C., Kranenburg, T. A., Hogg, T., Poppleton, H., Martin, J. et al. (2010). Subtypes of medulloblastoma have distinct developmental origins. *Nature* 468, 1095-1099. 10.1038/nature09587 21150899 PMC3059767

[JCS258608C23] Grimmer, M. R. and Weiss, W. A. (2008). BMPs oppose Math1 in cerebellar development and in medulloblastoma. *Genes Dev.* 22, 693-699. 10.1101/gad.1657808 18347086 PMC2731664

[JCS258608C24] Haldipur, P., Bharti, U., Govindan, S., Sarkar, C., Iyengar, S., Gressens, P. and Mani, S. (2012). Expression of sonic hedgehog during cell proliferation in the human cerebellum. *Stem Cells Dev.* 21, 1059-1068. 10.1089/scd.2011.0206 21732818

[JCS258608C25] Han, Y. G., Kim, H. J., Dlugosz, A. A., Ellison, D. W., Gilbertson, R. J. and Alvarez-Buylla, A. (2009). Dual and opposing roles of primary cilia in medulloblastoma development. *Nat. Med.* 15, 1062-1065. 10.1038/nm.2020 19701203 PMC2771737

[JCS258608C26] Hassounah, N. B., Bunch, T. A. and McDermott, K. M. (2012). Molecular pathways: The role of primary cilia in cancer progression and therapeutics with a focus on hedgehog signaling. *Clin. Cancer Res.* 18, 2429-2435. 10.1158/1078-0432.CCR-11-0755 22415315 PMC3738179

[JCS258608C27] Hatten, M. E. (1985). Neuronal regulation of astroglial morphology and proliferation in vitro. *J. Cell Biol.* 100, 384-396. 10.1083/jcb.100.2.384 3881455 PMC2113456

[JCS258608C28] Hatten, M. E. and Roussel, M. F. (2011). Development and cancer of the cerebellum. *Trends Neurosci.* 34, 134-142. 10.1016/j.tins.2011.01.002 21315459 PMC3051031

[JCS258608C29] Haycraft, C. J., Banizs, B., Aydin-Son, Y., Zhang, Q., Michaud, E. J. and Yoder, B. K. (2005). Gli2 and Gli3 localize to cilia and require the intraflagellar transport protein polaris for processing and function. *PLoS Genet.* 1, e53. 10.1371/journal.pgen.0010053 16254602 PMC1270009

[JCS258608C30] Hovestadt, V., Smith, K. S., Bihannic, L., Filbin, M. G., Shaw, M. L., Baumgartner, A., DeWitt, J. C., Groves, A., Mayr, L., Weisman, H. R. et al. (2019). Resolving medulloblastoma cellular architecture by single-cell genomics. *Nature* 572, 74-79. 10.1038/s41586-019-1434-6 31341285 PMC6754173

[JCS258608C31] Huang, D. W., Sherman, B. T., Lempicki, R. A., Huang, D., Sherman, B., Lempicki, R., Huang, D., Sherman, B., Lempicki, R., Maere, S. et al. (2009). Bioinformatics enrichment tools: paths toward the comprehensive functional analysis of large gene lists. *Nucleic Acids Res.* 37, 1-13. 10.1093/nar/gkn923 19033363 PMC2615629

[JCS258608C32] Huangfu, D. and Anderson, K. V. (2005). Cilia and Hedgehog responsiveness in the mouse. *Proc. Natl. Acad. Sci. USA* 102, 11325-11330. 10.1073/pnas.0505328102 16061793 PMC1183606

[JCS258608C33] Huangfu, D., Liu, A., Rakeman, A. S., Murcia, N. S., Niswander, L. and Anderson, K. V. (2003). Hedgehog signalling in the mouse requires intraflagellar transport proteins. *Nature* 426, 83-87. 10.1038/nature02061 14603322

[JCS258608C34] Jessa, S., Blanchet-Cohen, A., Krug, B., Vladoiu, M., Coutelier, M., Faury, D., Poreau, B., De Jay, N., Hébert, S., Monlong, J. et al. (2019). Stalled developmental programs at the root of pediatric brain tumors. *Nat. Genet* 51, 1702-1713. 31768071 10.1038/s41588-019-0531-7 PMC6885128

[JCS258608C35] Jia, J., Kolterud, Å., Zeng, H., Hoover, A., Teglund, S., Toftgård, R. and Liu, A. (2009). Suppressor of Fused inhibits mammalian Hedgehog signaling in the absence of cilia. *Dev. Biol.* 330, 452-460. 10.1016/j.ydbio.2009.04.009 19371734 PMC2687323

[JCS258608C36] Jiang, J. and Hui, C. (2008). Hedgehog signaling in development and cancer. *Dev. Cell* 15, 801-812. 10.1016/j.devcel.2008.11.010 19081070 PMC6443374

[JCS258608C37] Kenney, A. M. (2003). Nmyc upregulation by sonic hedgehog signaling promotes proliferation in developing cerebellar granule neuron precursors. *Development* 130, 15-28. 10.1242/dev.00182 12441288

[JCS258608C38] Kho, A. T., Zhao, Q., Cai, Z., Butte, A. J., Kim, J. Y. H., Pomeroy, S. L., Rowitch, D. H. and Kohane, I. S. (2004). Conserved mechanisms across development and tumorigenesis revealed by a mouse development perspective of human cancers. *Genes Dev.* 18, 629-640. 10.1101/gad.1182504 15075291 PMC387239

[JCS258608C39] Kim, J. J., Gill, P. S., Rotin, L., van Eede, M., Henkelman, R. M., Hui, C.-C. and Rosenblum, N. D. (2011). Suppressor of fused controls mid-hindbrain patterning and cerebellar morphogenesis via GLI3 repressor. *J. Neurosci.* 31, 1825-1836. 10.1523/JNEUROSCI.2166-10.2011 21289193 PMC6623745

[JCS258608C40] Knoepfler, P. S., Cheng, P. F. and Eisenman, R. N. (2002). N-myc is essential during neurogenesis for the rapid expansion of progenitor cell populations and the inhibition of neuronal differentiation. *Genes Dev.* 16, 2699-2712. 10.1101/gad.1021202 12381668 PMC187459

[JCS258608C41] Kool, M., Jones, D. T. W., Jäger, N., Northcott, P. A., Pugh, T. J., Hovestadt, V., Piro, R. M., Esparza, L. A., Markant, S. L., Remke, M. et al. (2014). Genome sequencing of SHH medulloblastoma predicts genotype-related response to smoothened inhibition. *Cancer Cell* 25, 393-405. 10.1016/j.ccr.2014.02.004 24651015 PMC4493053

[JCS258608C42] Korthuis, P. M., Berger, G., Bakker, B., Rozenveld-Geugien, M., Jaques, J., De Haan, G., Schuringa, J. J., Vellenga, E. and Schepers, H. (2015). CITED2-mediated human hematopoietic stem cell maintenance is critical for acute myeloid leukemia. *Leukemia* 29, 625-635. 10.1038/leu.2014.259 25184385

[JCS258608C43] Lafranchi, L., de Boer, H. R., de Vries, E. G., Ong, S., Sartori, A. A. and van Vugt, M. A. (2014). APC/C ^C dh1^ controls CtIP stability during the cell cycle and in response to DNA damage. *EMBO J.* 33, 2860-2879. 10.15252/embj.201489017 25349192 PMC4282561

[JCS258608C44] Lee, H. Y., Greene, L. A., Mason, C. A. and Manzini, M. C. (2009). Isolation and culture of post-natal mouse cerebellar granule neuron progenitor cells and neurons. *J. Vis. Exp* 23, 990. 10.3791/990 PMC2781826 19229177

[JCS258608C45] Leto, K., Arancillo, M., Becker, E. B. E., Buffo, A., Chiang, C., Ding, B., Dobyns, W. B., Dusart, I., Haldipur, P., Hatten, M. E. et al. (2016). Consensus paper: cerebellar development. *The Cerebellum* 15, 789-828. 10.1007/s12311-015-0724-2 26439486 PMC4846577

[JCS258608C46] Lewis, P. M., Gritli-Linde, A., Smeyne, R., Kottmann, A. and McMahon, A. P. (2004). Sonic hedgehog signaling is required for expansion of granule neuron precursors and patterning of the mouse cerebellum. *Dev. Biol.* 270, 393-410. 10.1016/j.ydbio.2004.03.007 15183722

[JCS258608C47] Lin, C. Y., Erkek, S., Tong, Y., Yin, L., Federation, A. J., Zapatka, M., Haldipur, P., Kawauchi, D., Risch, T., Warnatz, H. J. et al. (2016). Active medulloblastoma enhancers reveal subgroup-specific cellular origins. *Nature* 530, 57-62. 10.1038/nature16546 26814967 PMC5168934

[JCS258608C48] MacHold, R. and Fishell, G. (2005). Math1 is expressed in temporally discrete pools of cerebellar rhombic-lip neural progenitors. *Neuron* 48, 17-24. 10.1016/j.neuron.2005.08.028 16202705

[JCS258608C49] Machold, R., Klein, C. and Fishell, G. (2011). Genes expressed in Atoh1 neuronal lineages arising from the r1/isthmus rhombic lip. *Gene Expr. Patterns* 11, 349-359. 10.1016/j.gep.2011.03.007 21440680 PMC3095718

[JCS258608C50] Madisen, L., Zwingman, T. A., Sunkin, S. M., Oh, S. W., Zariwala, H. A., Gu, H., Ng, L. L., Palmiter, R. D., Hawrylycz, M. J., Jones, A. R. et al. (2010). A robust and high-throughput Cre reporting and characterization system for the whole mouse brain. *Nat. Neurosci.* 13, 133-140. 10.1038/nn.2467 20023653 PMC2840225

[JCS258608C51] May, S. R., Ashique, A. M., Karlen, M., Wang, B., Shen, Y., Zarbalis, K., Reiter, J., Ericson, J. and Peterson, A. S. (2005). Loss of the retrograde motor for IFT disrupts localization of Smo to cilia and prevents the expression of both activator and repressor functions of Gli. *Dev. Biol.* 287, 378-389. 10.1016/j.ydbio.2005.08.050 16229832

[JCS258608C52] Merico, D., Isserlin, R., Stueker, O., Emili, A. and Bader, G. D. (2010). Enrichment map: a network-based method for gene-set enrichment visualization and interpretation. *PLoS One* 5, e13984. 10.1371/journal.pone.0013984 21085593 PMC2981572

[JCS258608C53] Miale, I. L. and Sidman, R. L. (1961). An autoradiographic analysis of histogenesis in the mouse cerebellum. *Exp. Neurol* 4, 277-296. 10.1016/0014-4886(61)90055-3 14473282

[JCS258608C54] Michaud, E. J. and Yoder, B. K. (2006). The primary cilium in cell signaling and cancer. *Cancer Res.* 66, 6463-6467. 10.1158/0008-5472.CAN-06-0462 16818613

[JCS258608C55] Monnier, V., Dussillol, F., Alves, G., Lamour-Isnard, C. and Plessis, A. (1998). Suppressor of fused links fused and Cubitus interruptus on the hedgehog signalling pathway. *Curr. Biol.* 8, 583-586. 10.1016/S0960-9822(98)70227-1 9601642

[JCS258608C56] Northcott, P. A., Hielscher, T., Dubuc, A., MacK, S., Shih, D., Remke, M., Al-Halabi, H., Albrecht, S., Jabado, N., Eberhart, C. G. et al. (2011). Pediatric and adult sonic hedgehog medulloblastomas are clinically and molecularly distinct. *Acta Neuropathol.* 122, 231-240. 10.1007/s00401-011-0846-7 21681522 PMC4538327

[JCS258608C57] Northcott, P. A., Jones, D. T. W., Kool, M., Robinson, G. W., Gilbertson, R. J., Cho, Y.-J., Pomeroy, S. L., Korshunov, A., Lichter, P., Taylor, M. D. et al. (2012a). Medulloblastomics: the end of the beginning. *Nat. Rev. Cancer* 12, 818-834. 10.1038/nrc3410 23175120 PMC3889646

[JCS258608C58] Northcott, P. A., Shih, D. J. H., Peacock, J., Garzia, L., Sorana Morrissy, A., Zichner, T., Stütz, A. M., Korshunov, A., Reimand, J., Schumacher, S. E. et al. (2012b). Subgroup-specific structural variation across 1,000 medulloblastoma genomes. *Nature* 488, 49-56. 10.1038/nature11327 22832581 PMC3683624

[JCS258608C59] Northcott, P. A., Buchhalter, I., Morrissy, A. S., Hovestadt, V., Weischenfeldt, J., Ehrenberger, T., Gröbner, S., Segura-Wang, M., Zichner, T., Rudneva, V. A. et al. (2017). The whole-genome landscape of medulloblastoma subtypes. *Nature* 547, 311-317. 10.1038/nature22973 28726821 PMC5905700

[JCS258608C60] Ohli, J., Neumann, J. E., Grammel, D. and Schüller, U. (2015). Localization of SHH medulloblastoma in mice depends on the age at its initiation. *Acta Neuropathol.* 130, 307-309. 10.1007/s00401-015-1453-9 26092032

[JCS258608C61] Ong, T., Trivedi, N., Wakefield, R., Frase, S. and Solecki, D. J. (2020). Siah2 integrates mitogenic and extracellular matrix signals linking neuronal progenitor ciliogenesis with germinal zone occupancy. *Nat. Commun.* 11, 5312. 10.1038/s41467-020-19063-7 33082319 PMC7576183

[JCS258608C62] Pearse, R. V., Collier, L. S., Scott, M. P. and Tabin, C. J. (1999). Vertebrate homologs of Drosophila Suppressor of fused interact with the Gli family of transcriptional regulators. *Dev. Biol.* 212, 323-336. 10.1006/dbio.1999.9335 10433824 PMC4530617

[JCS258608C63] Pöschl, J., Stark, S., Neumann, P., Gröbner, S., Kawauchi, D., Jones, D. T. W., Northcott, P. A., Lichter, P., Pfister, S. M., Kool, M. et al. (2014). Genomic and transcriptomic analyses match medulloblastoma mouse models to their human counterparts. *Acta Neuropathol.* 128, 123-136. 10.1007/s00401-014-1297-8 24871706

[JCS258608C64] Rausch, T., Jones, D. T. W., Zapatka, M., Stütz, A. M., Zichner, T., Weischenfeldt, J., Jäger, N., Remke, M., Shih, D., Northcott, P. A. et al. (2012). Genome sequencing of pediatric medulloblastoma links catastrophic DNA rearrangements with TP53 mutations. *Cell* 148, 59-71. 10.1016/j.cell.2011.12.013 22265402 PMC3332216

[JCS258608C65] Rohatgi, R., Milenkovic, L. and Scott, M. P. (2007). Patched1 regulates hedgehog signaling at the primary cilium. *Science (80–.)* 317, 372-376. 10.1126/science.1139740 17641202

[JCS258608C92] Robinson, M. D., McCarthy, D. J. and Smyth, G. K. (2010). edgeR: a Bioconductor package for differential expression analysis of digital gene expression data. *Bioinformatics*, 26, 139-140. 10.1093/bioinformatics/btp616 19910308 PMC2796818

[JCS258608C66] Roussel, M. F. and Stripay, J. L. (2018). Epigenetic drivers in pediatric medulloblastoma. *Cerebellum* 17, 28-36. 10.1007/s12311-017-0899-9 29178021 PMC5807456

[JCS258608C67] Salero, E. and Hatten, M. E. (2007). Differentiation of ES cells into cerebellar neurons. *Proc. Natl. Acad. Sci. USA* 104, 2997-3002. 10.1073/pnas.0610879104 17293457 PMC1796781

[JCS258608C68] Salsano, E., Pollo, B., Eoli, M., Giordana, M. T. and Finocchiaro, G. (2004). Expression of MATH1, a marker of cerebellar granule cell progenitors, identifies different medulloblastoma sub-types. *Neurosci. Lett* 370, 180-185. 10.1016/j.neulet.2004.08.053 15488319

[JCS258608C69] Sasai, N. and Briscoe, J. (2012). Primary cilia and graded Sonic Hedgehog signaling. *Wiley Interdiscip. Rev.* 1, 753-772. 10.1002/wdev.43 23799571

[JCS258608C70] Schindelin, J., Arganda-Carreras, I., Frise, E., Kaynig, V., Longair, M., Pietzsch, T., Preibisch, S., Rueden, C., Saalfeld, S., Schmid, B. et al. (2012). Fiji: An open-source platform for biological-image analysis. *Nat. Methods* 9, 676-682. 10.1038/nmeth.2019 22743772 PMC3855844

[JCS258608C71] Schüller, U., Heine, V. M., Mao, J., Kho, A. T., Dillon, A. K., Han, Y. G., Huillard, E., Sun, T., Ligon, A. H., Qian, Y. et al. (2008). Acquisition of granule neuron precursor identity is a critical determinant of progenitor cell competence to form Shh-induced medulloblastoma. *Cancer Cell* 14, 123-134. 10.1016/j.ccr.2008.07.005 18691547 PMC2597270

[JCS258608C72] Selvadurai, H. J., Luis, E., Desai, K., Lan, X., Vladoiu, M. C., Whitley, O., Galvin, C., Vanner, R. J., Lee, L., Whetstone, H. et al. (2020). Medulloblastoma arises from the persistence of a rare and transient Sox2+ granule neuron precursor. *Cell Rep.* 31, 107511. 32294450 10.1016/j.celrep.2020.03.075

[JCS258608C73] Shannon, P., Markiel, A., Ozier, O., Baliga, N. S., Wang, J. T., Ramage, D., Amin, N., Schwikowski, B. and Ideker, T. (2003). Cytoscape: A software Environment for integrated models of biomolecular interaction networks. *Genome Res.* 13, 2498-2504. 10.1101/gr.1239303 14597658 PMC403769

[JCS258608C74] Sharpe, H. J., Pau, G., Dijkgraaf, G. J., Basset-Seguin, N., Modrusan, Z., Januario, T., Tsui, V., Durham, A. B., Dlugosz, A. A., Haverty, P. M. et al. (2015). Genomic analysis of smoothened inhibitor resistance in basal cell carcinoma. *Cancer Cell* 27, 327-341. 10.1016/j.ccell.2015.02.001 25759019 PMC5675004

[JCS258608C75] Spassky, N., Han, Y. G., Aguilar, A., Strehl, L., Besse, L., Laclef, C., Romaguera Ros, M., Garcia-Verdugo, J. M. and Alvarez-Buylla, A. (2008). Primary cilia are required for cerebellar development and Shh-dependent expansion of progenitor pool. *Dev. Biol.* 317, 246-259. 10.1016/j.ydbio.2008.02.026 18353302 PMC4043448

[JCS258608C76] Svärd, J., Henricson, K. H., Persson-Lek, M., Rozell, B., Lauth, M., Bergström, Å., Ericson, J., Toftgård, R. and Teglund, S. (2006). Genetic elimination of suppressor of fused reveals an essential repressor function in the mammalian hedgehog signaling pathway. *Dev. Cell* 10, 187-197. 10.1016/j.devcel.2005.12.013 16459298

[JCS258608C77] Swartling, F. J., Savov, V., Persson, A. I., Chen, J., Hackett, C. S., Northcott, P. A., Grimmer, M. R., Lau, J., Chesler, L., Perry, A. et al. (2012). Distinct neural stem cell populations give rise to disparate brain tumors in response to N-MYC. *Cancer Cell* 21, 601-613. 10.1016/j.ccr.2012.04.012 22624711 PMC3360885

[JCS258608C78] Taipale, J., Chen, J. K., Cooper, M. K., Wang, B., Mann, R. K., Milenkovic, L., Scott, M. P. and Beachy, P. A. (2000). Effects of oncogenic mutations in Smoothened and Patched can be reversed by cyclopamine. *Nature* 406, 1005-1009. 10.1038/35023008 10984056

[JCS258608C79] Tan, I.-L., Wojcinski, A., Rallapalli, H., Lao, Z., Sanghrajka, R. M., Stephen, D., Volkova, E., Korshunov, A., Remke, M., Taylor, M. D. et al. (2018). Lateral cerebellum is preferentially sensitive to high sonic hedgehog signaling and medulloblastoma formation. *Proc. Natl. Acad. Sci USA* 115, 3392-3397. 10.1073/pnas.1717815115 29531057 PMC5879676

[JCS258608C80] Varjosalo, M., Li, S. P. and Taipale, J. (2006). Divergence of hedgehog signal transduction mechanism between Drosophila and mammals. *Dev. Cell* 10, 177-186. 10.1016/j.devcel.2005.12.014 16459297

[JCS258608C81] Vladoiu, M. C., El-Hamamy, I., Donovan, L. K., Farooq, H., Holgado, B. L., Sundaravadanam, Y., Ramaswamy, V., Hendrikse, L. D., Kumar, S., Mack, S. C. et al. (2019). Childhood cerebellar tumours mirror conserved fetal transcriptional programs. *Nature* 572, 67-73. 10.1038/s41586-019-1158-7 31043743 PMC6675628

[JCS258608C82] Wallace, V. A. (1999). Purkinje-cell-derived Sonic hedgehog regulates granule neuron precursor cell proliferation in the developing mouse cerebellum. *Curr. Biol* 9, 445-448. 10.1016/S0960-9822(99)80195-X 10226030

[JCS258608C83] Wang, V. Y., Rose, M. F. and Zoghbi, H. Y. (2005). Math1 expression redefines the rhombic lip derivatives and reveals novel lineages within the brainstem and cerebellum. *Neuron* 48, 31-43. 10.1016/j.neuron.2005.08.024 16202707

[JCS258608C84] Wechsler-Reya, R. J. and Scott, M. P. (1999). Control of neuronal precursor proliferation in the cerebellum by sonic hedgehog. *Neuron* 22, 103-114. 10.1016/S0896-6273(00)80682-0 10027293

[JCS258608C85] Wefers, A. K., Warmuth-Metz, M., Pöschl, J., Von Bueren, A. O., Monoranu, C. M., Seelos, K., Peraud, A., Tonn, J. C., Koch, A., Pietsch, T. et al. (2014). Subgroup-specific localization of human medulloblastoma based on pre-operative MRI. *Acta Neuropathol.* 127, 931-933. 10.1007/s00401-014-1271-5 24699697

[JCS258608C86] Wheway, G., Nazlamova, L. and Hancock, J. T. (2018). Signaling through the primary cilium. *Front. Cell Dev. Biol.* 6, 8. 10.3389/fcell.2018.00008 29473038 PMC5809511

[JCS258608C87] Yang, Z. J., Ellis, T., Markant, S. L., Read, T. A., Kessler, J. D., Bourboulas, M., Schüller, U., Machold, R., Fishell, G., Rowitch, D. H. et al. (2008). Medulloblastoma can be initiated by deletion of patched in lineage-restricted progenitors or stem cells. *Cancer Cell* 14, 135-145. 10.1016/j.ccr.2008.07.003 18691548 PMC2538687

[JCS258608C88] Yokota, N., Aruga, J., Takai, S., Yamada, K., Hamazaki, M., Iwase, T., Sugimura, H. and Mikoshiba, K. (1996). Predominant expression of human Zic in cerebellar granule cell lineage and medulloblastoma. *Cancer Res.* 56, 377-383. 8542595

[JCS258608C89] Zhao, X., Pak, E., Ornell, K. J., Murphy, M. F. P., Mackenzie, E. L., Chadwick, E. J., Ponomaryov, T., Kelleher, J. F. and Segal, R. A. (2017). A transposon screen identifies loss of primary cilia as a mechanism of resistance to SMO inhibitors. *Cancer Discov.* 7, 1436-1439. 10.1158/2159-8290.CD-17-0281 28923910

[JCS258608C90] Zhu, X., Girardo, D., Govek, E. E., John, K., Mellén, M., Tamayo, P., Mesirov, J. P. and Hatten, M. E. (2016). Role of Tet1/3 genes and chromatin remodeling genes in cerebellar circuit formation. *Neuron* 89, 100-112. 10.1016/j.neuron.2015.11.030 26711116 PMC4707072

[JCS258608C91] Zomerman, W. W., Plasschaert, S. L. A., Conroy, S., Scherpen, F. J., Meeuwsen-de Boer, T. G. J., Lourens, H. J., Guerrero Llobet, S., Smit, M. J., Slagter-Menkema, L., Seitz, A. et al. (2018). Identification of two protein-signaling states delineating transcriptionally heterogeneous human medulloblastoma. *Cell Rep.* 22, 3206-3216. 10.1016/j.celrep.2018.02.089 29562177

