# Supplementary material for: The developmental stage of the medulloblastoma cell-of-origin restricts Sonic hedgehog pathway usage and drug sensitivity
Source: J Cell Sci. 2022 Jun 8;135(11):jeb258608. doi: 10.1242/jcs.258608 (PMC9234672; doi:10.1242/jcs.258608)
Supplement: Supplementary information [file joces-135-258608-s1.pdf]

Figure S1 Related to Figure 2

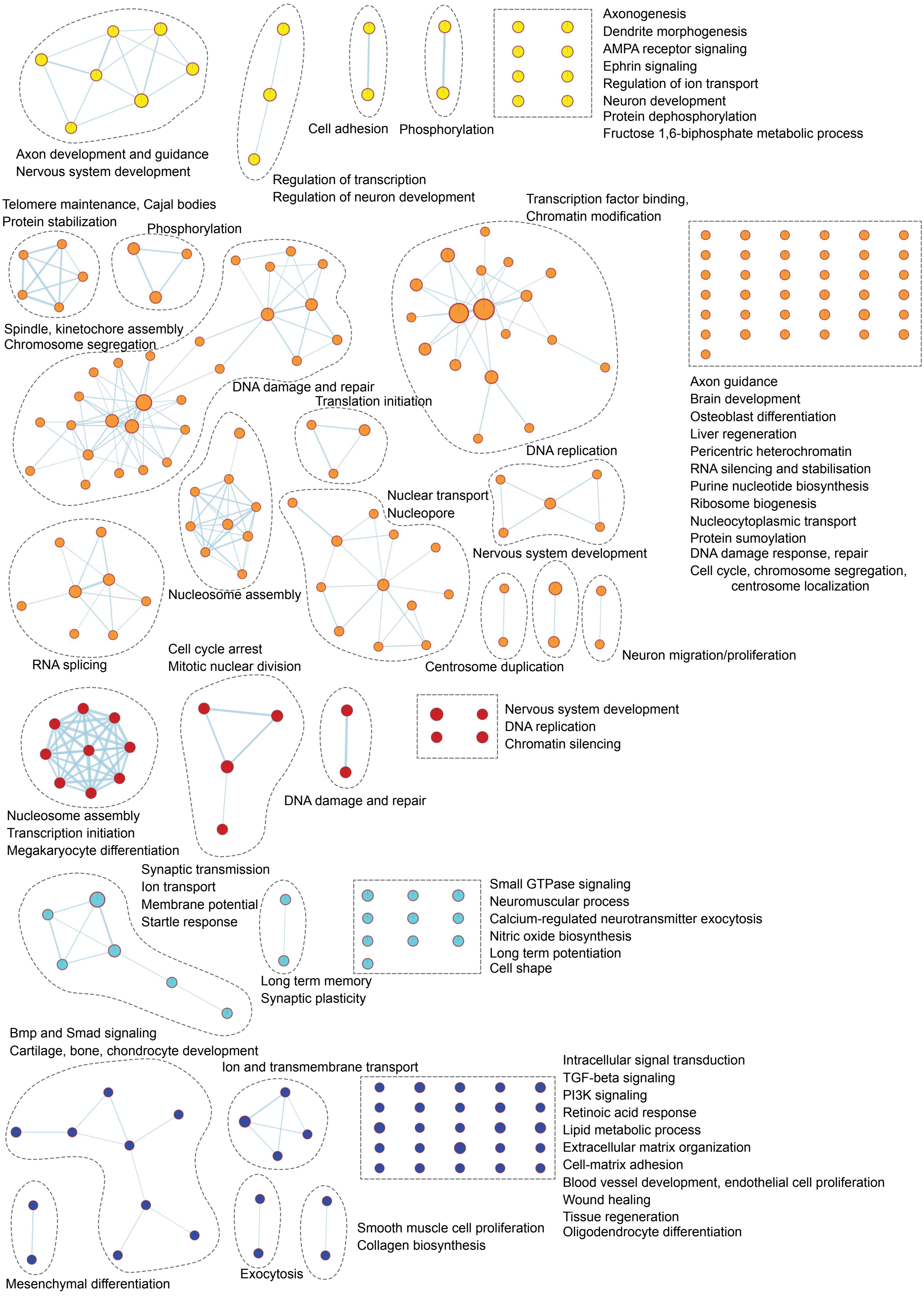

Journal of Cell Science • Supplementary information

Fig. S1, related to Figure 2.

Enrichment of specific biological processes during CGNP development per gene cluster. Gene ontological analysis shows enriched biological processes per gene cluster. Each node represents a biological process. Related biological processes are grouped and labeled by biological theme (curved dashed lines). Individual biological processes are assembled in rectangular boxes (dashed lines). Biological processes connected by edges have genes in common. Enriched biological processes were determined with the Database of Annotation, Visualization and Integrated Discovery (DAVID), v.6.8 (Benjamini-corrected q = 0.1, p =0.01) and visualized with the Enrichment Map app in Cytoscape. Yellow nodes: E15.5 - E17.5 cluster; orange nodes: E15.5 - P7 clusters; red nodes: P0 - P7 cluster; light blue nodes: P14 - P30 cluster; dark blue nodes: P14 - P30 cluster.

Figure S2 Related to Figure 3

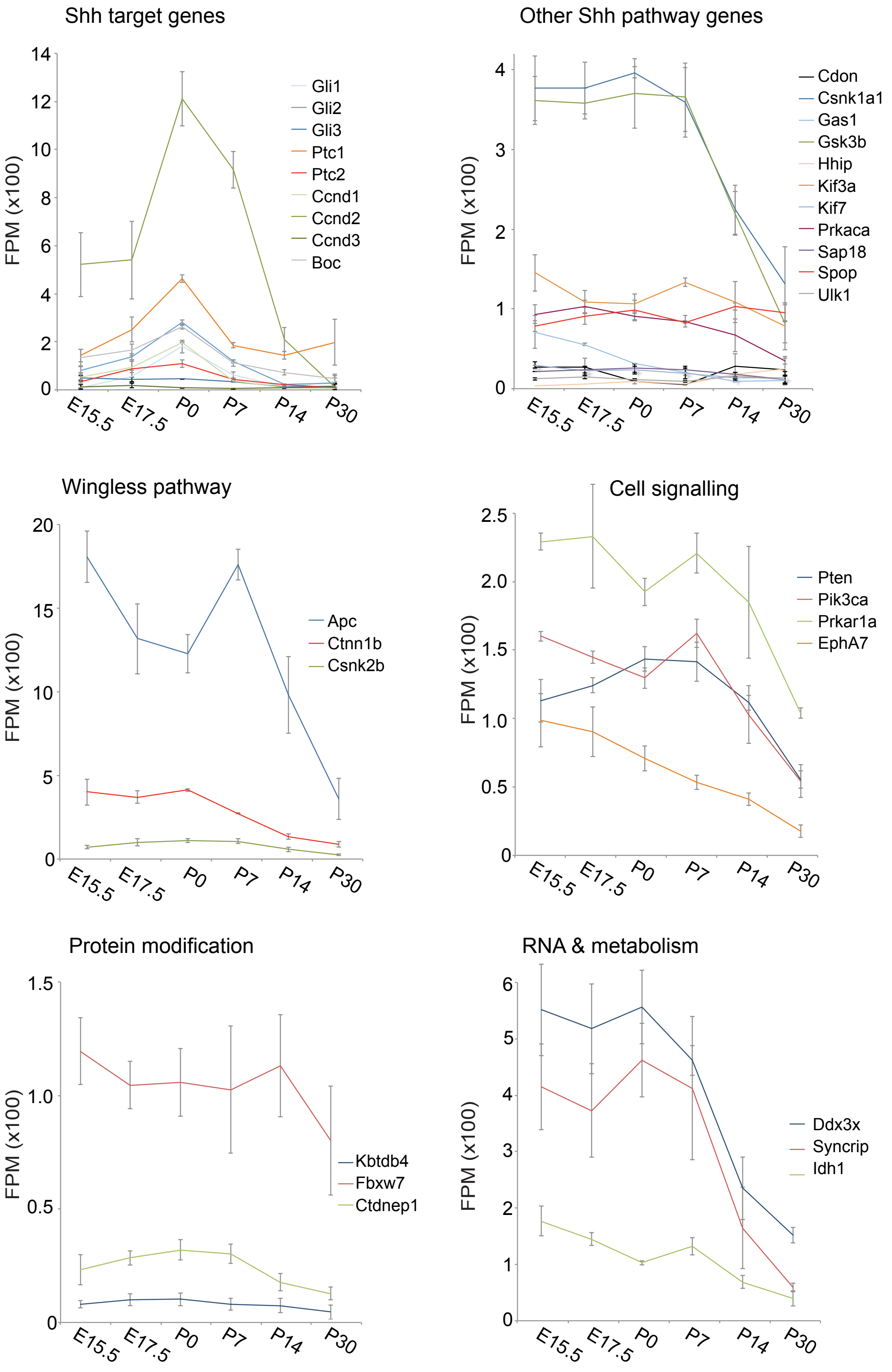

Journal of Cell Science • Supplementary information

Fig. S2, related to Figure 3.

Gene expression and proliferation in developing CGNPs.

Gene expression profiles of CGNP genes commonly mutated in medulloblastoma extracted from the RNA-seq data set. Curves represent the average expression level from three biological replicates, error bars indicate standard deviation (FPM=fragments per million).

Figure S3 Related to Figures 5-6

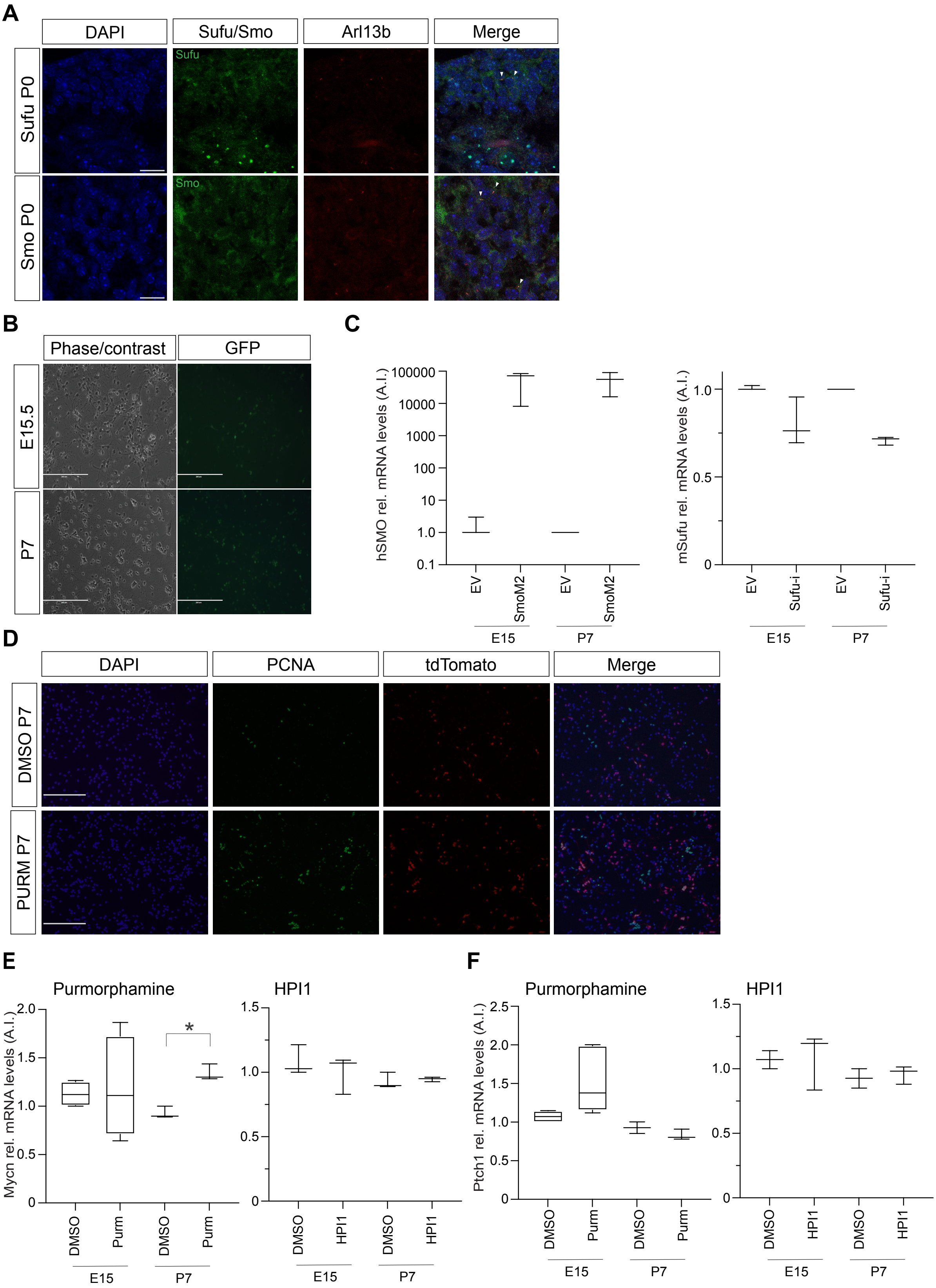

Journal of Cell Science • Supplementary information

Fig. S3, related to Figures 5-6.

Primary cilia expression in developing cerebellum and lentiviral transduction, proliferation and gene expression in primary CGNPs.

(A) Confocal images showing Sufu (upper panels) or Smo (lower panels), and Arl13b protein expression in the P0 mouse cerebellum. Arrow heads indicate colocalization of Sufu or Smo with Arl13b (P, postnatal day; EGL, external granular layer; IGL, internal granular layer). Scale bars, 10 μm.

(B) Phase contrast images (left panels) and GFP fluorescence (right panels) showing representative lentiviral transduction efficiency in E15.5 and P7 CGNPs. Scale bars, 200 μm.

(C) Box and whisker plots showing relative hSMO (left chart) and mSufu (right chart) mRNA expression levels compared to Gapdh as determined by qRT-PCR, in either SmoM2 or Sufu-i shRNA versus empty vector control (EV) transduced E15.5 and P7 CGNPs. n=3 biological replicates Whiskers represent min to max. n=3 biological replicates (A.I., arbitrary units).

(D) Representative immunofluorescent images of P7 primary CGNP cultures immunolabeled for PCNA and tdTomato after 48 hrs treatment with Purmorphamine (Purm) or DMSO. Scale bars, 200 μm.

(E) Box and whisker plots showing relative Mycn mRNA expression levels compared to Gapdh as determined by qRT-PCR, in either Purmorphamine (left chart) or HPI1 (right chart) versus DMSO treated E15.5 and P7 CGNPs. Whiskers represent min to max. E15 Purmorphamine, n=5; P7 Purmorphamine, n=3; E15 HPI1, n=3; and P7 HPI1, n=3 biological replicates (A.I., arbitrary units).

(F) Box and whisker plots showing relative Ptch1 mRNA expression levels compared to Gapdh as determined by qRT-PCR, in either Purmorphamine (left chart) or HPI1 (right chart) versus DMSO treated E15.5 and P7 CGNPs. Whiskers represent min to max. E15 Purmorphamine, n=5; P7 Purmorphamine, n=3; E15 HPI1, n=3; and P7 HPI1, n=3 biological replicates (A.I., arbitrary units).

Journal of Cell Science • Supplementary information

Table S1, related to Figure 1. Raw counts CGNP RNA-seq.

Click here to download Table S1

Table S2, related to Figure 1. Gene clusters (CGNP RNA-seq).

Click here to download Table S2

Table S3, related to Figure 2. Biological processes (CGNP RNA-seq).

Click here to download Table S3

Table S4, related to Figure 3. Upper and lower gene clusters (cross species comparison).

Click here to download Table S4

Table S5, related to Figure 3. Biological processes (cross species comparison).

Click here to download Table S5

Journal of Cell Science • Supplementary information

Table S6, related to Materials and methods

**Primers qRT-PCR**	**Sequence**
mGapdh Forward	5'-AGGGCTCATGACCACAGTC-3'
mGapdh Reverse	5'-GATGCAGGGATGATGTTCTG-3'
hSMO Forward	5'-TGAAGGCTGCACGAATGAGG-3'
hSMO Reverse	5'-CTTGGGGTTGTCTGTCCGAA-3'
mSufu Forward	5'-ACATCAGCTTTGGCCTGAGT-3'
mSufu Reverse	5'-AAATCCACTTGGTCCGTCTG-3'
mGli1 Forward	5'-GGTCTCGGGGTCTCAAACTGC-3'
mGli1 Reverse	5'-CGGCTGACTGTGTAAGCAGAG-3'
mNmyc Forward	5'-GTCTTCCCCTTCCCGGTGAAC-3'
mNmyc Reverse	5'-CAAGGTATCCTCTCCGGAGGTGC-3'
mPtch Forward	5'-ATTGCATCTGTTGGCATCGG-3'
mPtch Reverse	5'-AGAACGGGAGCAAACATGTG-3'
mCcnd1 Forward	5'-AGACCTGTGCGCCCTCCGTA-3'
mCcnd1 Reverse	5'-CAGCTGCAGGCGGCTCTTCT-3'
mCcnd2 Forward	5'-CTGTGCGCTACCGACTTCAA-3'
mCcnd2 Reverse	5'-ATCATCCTGCTGAAGCCCAC-3'
**Oligo siRNA**	**Sequence**
Sufu A	5'-TTGAGTTGACGTTTCGTCTGAA-3'
**Primers (cloning)**	**Sequence**
miRE-XhoI Forward	5'-TGAACTCGAGAAGGTATATTGCTGTTGACAGTGAGCG-'3
miRE-EcoRI Reverse	5'-TCTCGAATTCTAGCCCCTTGAAGTCCGAGGCAGTAGGC-3'

Journal of Cell Science • Supplementary information
